# Automated Filtering of Intrinsic Movement Artifacts during Two-Photon Intravital Microscopy

**DOI:** 10.1371/journal.pone.0053942

**Published:** 2013-01-11

**Authors:** Denis Soulet, Alexandre Paré, Julien Coste, Steve Lacroix

**Affiliations:** 1 Centre de recherche du CHUQ-CHUL, Axe neurosciences, Québec, Canada; 2 Département de psychiatrie et neurosciences, Faculté de médecine, Université Laval, Québec, Canada; 3 Département de médecine moléculaire, Faculté de médecine, Université Laval, Québec, Canada; University G. D’Annunzio, Italy

## Abstract

*In vivo* imaging using two-photon microscopy is an essential tool to explore the dynamic of physiological events deep within biological tissues for short or extended periods of time. The new capabilities offered by this technology (e.g. high tissue penetrance, low toxicity) have opened a whole new era of investigations in modern biomedical research. However, the potential of using this promising technique in tissues of living animals is greatly limited by the intrinsic irregular movements that are caused by cardiac and respiratory cycles and muscular and vascular tone. Here, we show real-time imaging of the brain, spinal cord, sciatic nerve and myenteric plexus of living mice using a new automated program, named Intravital_Microscopy_Toolbox, that removes frames corrupted with motion artifacts from time-lapse videos. Our approach involves generating a dissimilarity score against precalculated reference frames in a specific reference channel, thus allowing the gating of distorted, out-of-focus or translated frames. Since the algorithm detects the uneven peaks of image distortion caused by irregular animal movements, the macro allows a fast and efficient filtering of the image sequence. In addition, extra features have been implemented in the macro, such as XY registration, channel subtraction, extended field of view with maximum intensity projection, noise reduction with average intensity projections, and automated timestamp and scale bar overlay. Thus, the Intravital_Microscopy_Toolbox macro for ImageJ provides convenient tools for biologists who are performing *in vivo* two-photon imaging in tissues prone to motion artifacts.

## Introduction

Two-photon intravital microscopy (2P-IVM) has become a tool of choice to longitudinally explore dynamic physiological events within living tissues with minimal photobleaching and phototoxicity. This technology provides not only the ability to track cell movements [1], [2], but also to observe morphological changes in small cellular structures [3], [4], and even monitor cell proliferation and death [5]. 2P-IVM has already revolutionized all areas of biology [6], and yet we are only at the beginning of a new era in dynamic biological imaging.

One major limitation of 2P-IVM that has yet to be addressed is that image sequences are commonly plagued with motion artifacts caused by cardiac and respiratory cycles and muscular and vascular tone. These movements are especially present in body parts that cannot be completely restrained, even under deep anesthesia. To make things worst, animals anesthetized with isoflurane, a safe anesthetic that provides rapid induction and recovery, often do not breathe regularly, which impairs the possibility of triggering the acquisition following a constant frequency. Other methods that reduce the source of movements, such as endotracheal intubation for mechanical ventilation [7], [8], cardiopulmonary bypass [9], and injection of myorelaxants [10] or muscle paralyzing drugs [11], can physiologically affect the experiment and lead to death if not carefully monitored. Recently, Laffray and co-workers have developed an ingenious stabilization device, featuring an off-axis laser and a piezo nanopositioner, that constantly maintains the focus [12]. However, such a system is relatively costly and requires a custom installation. Here, we introduce a free public-domain software package that allows for automated processing and filtering of image sequences generated using 2P-IVM.

## Materials and Methods

### Ethics Statement

All animal manipulations in this study were reviewed and approved by the Laval University Animal Care Committee (approval 2009051-3 and 2012093-1) and in compliance with guidelines from the Canadian Council on Animal Care.

### Animals

Thy1-CFP-23 and CX_3_CR1-eGFP transgenic mice were purchased from The Jackson Laboratory (Bar Harbor, ME). LysM-eGFP breeders were obtained from Dr. Gregory Dekaban (The Robarts Institute, London, ON), after first obtaining authorization from the creator of this mouse, Dr. Thomas Graf (Barcelona, Spain). Mice were bred and cross-bred in-house at the Animal Research Facility of the CHUL Research Center and genotyped as described on The Jackson Laboratory website or according to the method published by Faust et al. [13]. All mice had free access to food and water.

### Animal Stabilization and Surgeries

#### In vivo imaging of the mouse brain (Figure S1a)

Mice (8–12 weeks old) were deeply anesthetized with isoflurane and mounted in a cranial stereotaxic apparatus (David Kopf Instruments, Tujunga, CA). An incision was made to expose the skull and a small cranial window drilled at coordinates A/P 0.83 mm, M/L 0.5 mm relative to the bregma. To avoid tissue dehydration and keep the water objective adequately immersed, a Gelseal™ (GE Healthcare, Baie d’Urfé, QC) pool was formed around the exposed area and filled with artificial cerebrospinal fluid (aCSF).

#### In vivo imaging of the mouse spinal cord (Figure S1b)

Anesthetized mice underwent a laminectomy at vertebral level T10 and the vertebral column stabilized at T9 and T11 using a custom-made stabilization device adapted for two-photon microscopy, and made of a steel base plate on which was mounted the David Kopf Instruments Mouse Nose/Tooth bar Assembly (model 926-B) and two pairs of stabilizing forceps attached to support arms that can be moved in the three directions. Once the vertebral column was suspended and aligned by means of the two vertebral forceps, the stabilization device was next placed on the stage right underneath the water immersion objective. As for the cranial window preparation, a Gelseal™ pool filled with aCSF was formed around the exposed spinal segments to immerse the objective lens. In the experiments in which chronic *in vivo* imaging of the spinal cord was necessary, an imaging chamber was installed following the method of Farrar et al. [14].

#### *In vivo* imaging of the mouse sciatic nerve (Figure S1c)

The sciatic nerve microcrush lesion was performed at mid-thigh level and the site of injury identified using a 10-0 Ethilon suture (Ethicon; Somerville, NJ) passed through the epineurium, following our previously published method [15], [16]. At specific time-points post-injury, mice were re-anesthetized with isoflurane, placed in a custom made constraining frame, and the leg of interest stretched using elastic bands connected to a magnetic retraction kit. The whole setup was then placed on the stage underneath the objective, and the position of the sciatic nerve aligned and adjusted to maximize the field of view and minimize intrinsic movements.

#### *In vivo* imaging of the mouse enteric nervous system (Figure S1d)

A 1.5 cm incision was made in the median line of the abdominal wall. The caecum was identified and gently pulled out of the peritoneal cavity. The proximal part of the caecum was brought out to expose the distal ileum. The animals were placed on the stage with the ileum exposed, which was then immobilized between a 20-mm-diameter stainless steel washer on the top and a wet gauze underneath. The hole of the washer was filled with isotonic saline, and a cover glass was placed on top. The whole setup was then placed on the stage underneath the objective, and the position of the ileum aligned and adjusted to maximize the field of view and minimize intrinsic movements.

For labeling of blood vessels, Texas red-dextran [17] (70 kDa, 1.25% w/v, Invitrogen Canada Inc., Mississauga, ON) or Qdot 705 (Qtracker® 705, 5% w/v in PBS, Invitrogen Canada Inc.) was injected into a tail vein 5 min prior the beginning of the imaging session. Body temperature was recorded through a rectal probe connected to a temperature controlling device (RWD Life Science Co., ShenZhen, GuangDong, China), and the temperature maintained at 37°C during all procedures. Following imaging, muscular layers were sutured and cutaneous layers stapled.

### Two-photon Laser Microscopy

All images were acquired on an Olympus FV1000 MPE two-photon microscope dedicated to intravital imaging and owned by the Lacroix laboratory. The two-photon Mai Tai DeepSee laser (Spectra-Physics, Newport Corp., Santa Clara, CA) was tuned at 950nm for all the experiments, and the output power was set between 14 and 84 mW. Tissues were imaged using an Olympus Ultra 25x MPE water immersion objective (1.05 NA), with filter set bandwidths optimized for CFP (460–500 nm), YFP (520–560 nm), Texas Red/DsRed (575–630 nm) and Qdot 705/800 (669–800 nm) imaging. Detector sensitivity and gain were set to achieve the optimal dynamic range of detection. Using the Olympus Fluoview software (version 3.0a), images with resolution ranging from 128×96 pixels to 512×512 pixels were acquired at different zoom factors (1× to 7×) and at 2.5 to 15 frames per second with auto-Hv option enabled, and exported as 24-bit RGB TIF files, while metadata for subsequent automatic setting-detection were exported in a TXT file. No Kalman filter was used to avoid slowing down the acquisition speed.

### Software Development

We have developed the “Intravital_Microscopy_Toolbox” macro using the 32-bit version of ImageJ 1.47a (Wayne Rasband, NIH, Bethesda) for Microsoft Windows, bundled with 32-bit Java 1.6.0_26. Acquired images were processed on a personal computer running Microsoft Windows Vista Ultimate 64-bit, Microsoft Windows 7 Premium Edition 64-bit. Maximum memory setting for ImageJ was set to 1200MB. Image-to-image alignment in the *x-y* plane was performed either using the StackReg and TurboReg plug-ins from P. Thévenaz, U.E. Ruttimann, M. Unser (École Polytechnique Fédérale de Lausanne, Switzerland), which was downloaded at http://bigwww.epfl.ch/thevenaz/stackreg/, or using the JavaSIFT plug-in from Stephan Saalfeld available at http://fly.mpi-cbg.de/~saalfeld/Projects/javasift.html. Subtle motion correction was performed using the Kalman_Stack_Filter plug-in from Christopher Philip Mauer, which was downloaded at http://rsbweb.nih.gov/ij/plugins/kalman.html. Code from the macro to generate the distribution plots has been derived from Tiago Ferreira’s code (http://imagejdocu.tudor.lu/doku.php?id=macro:distribution_plotter).

## Results

To test our macro, named Intravital_Microscopy_Toolbox, we performed 2P-IVM on various tissues of the nervous system (brain, spinal cord, sciatic nerve and myenteric plexus) both in naïve and injury/disease conditions. For that purpose, we used transgenic mice that express fluorescent proteins in a cell-lineage-specific manner (see **Materials and Methods**). We began by implementing new restraining techniques or ameliorated existing ones to minimize motion artifacts caused by animal breathing and other physiological movements (**Figure S1**). Although we achieved a major improvement in terms of stabilization, the images acquired were still prone to different kinds of motion artifacts (i.e. movements in the *x–y*, and/or *z* direction; see **Videos S1–S2**). Importantly, even the implantation of a spinal cord chamber, as recently developed by Farrar et al. [14], was associated with the presence of artifacts during 2P-IVM imaging (**Video S2, right panel**). Although the amplitude of movement in the *z* direction was significantly reduced with this method, it was sufficient to affect our ability to visualize fine movements of small cellular structures (e.g. motility of microglial processes). Thus, the animal models examined in this study were showing distinct motion artifacts, which we used to our advantage to develop and test the efficiency of the program on multichannel image sequences acquired using 2P-IVM.

**Figure 1:**
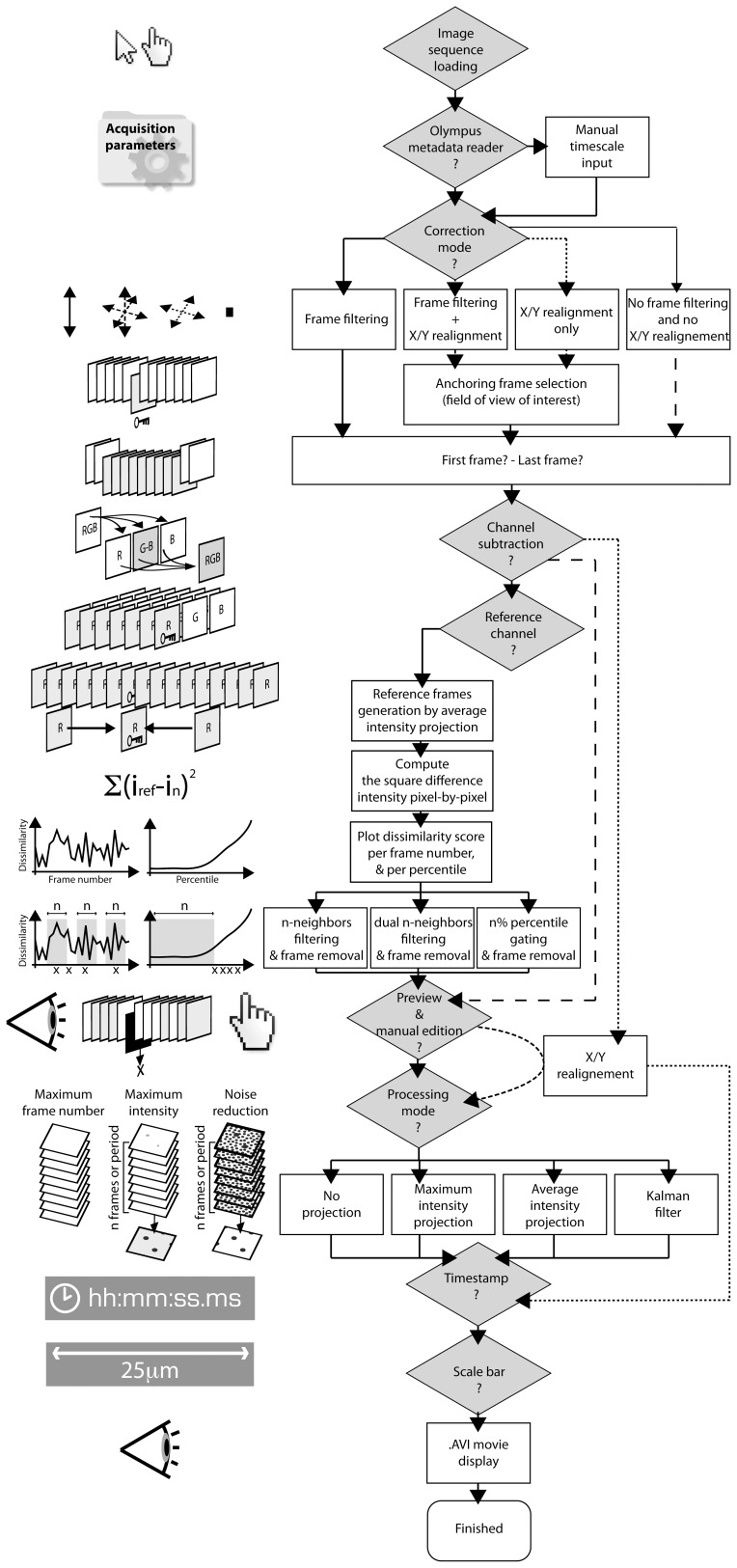
Macro flow chart for the “Intravital_Microscopy_Toolbox” program. Starting from the file selection, the image dissimilarity score is computed for each frame in the video. Main processing options are identified with grey shading.

The macro was designed to automatically generate reference frames, compute dissimilarity scores and remove distorted, translated, and out-of-focus images. To measure the presence of artifacts within the image sequence, we used a dissimilarity score calculated with a square difference algorithm against a reference image. In brief, the function slides through images, compares all the frames from the image sequence against a reference frame and stores dissimilarity scores in an array for subsequent gating using various filters (**Figure 1**). We intentionally avoided designs requiring command line inputs and opted to develop the program as a “one-click” macro for ImageJ, the most widely used public domain image processing software, usable by all. The macro was designed to handle 3 fluorescent imaging channels in simultaneous acquisition, and to sort images based on pixel-by-pixel comparisons using multiple reference frames. Because blood vessels are rarely subjected to rapid morphological changes, we used them as the reference channel to sort out images and remove artifactual frames.

To optimize the macro efficiency, we manually analyzed the original videos and qualitatively ranked each frame as either good, with minor artifacts or with major artifacts. We then tested two different approaches to calculate dissimilarity scores; one relying on the manual selection of a single reference frame (**Figs. S2, S3, S4, S5** and **Video S3**), and the other on the automated generation of groups of reference frames along the video sequence (**Figure S5** and **Videos S3–S4**), from now on referred to as automatic reference frame generation (RFG). For RFG, the macro was programmed to regroup images in series of *n* adjacent frames (e.g. series of 5–30 frames) centered on the image to be scored, and an average intensity projection calculated for each series, hence generating a reference frame. Each frame was then scored relative to its own reference frame. Our testing revealed that, although the single reference frame method allows to detect motion artifacts efficiently in short videos, the position of the reference frame within the image sequence had a profound effect on the dissimilarity scores and thus the filtering efficiency (**Figs. S2 and S5**), a problem that became even more noticeable in long videos (**Video S3**). As shown in **Table S1**, the presence of artifactual images and false positive and false negative rates were lower when using the automatic RFG compared with the single reference frame strategy. Furthermore, the baseline of the dissimilarity scores was more linear when the automatic RFG approach was used (**Figure S5**). For these reasons, all subsequent analyses were performed using the RFG method.

**Figure 2:**
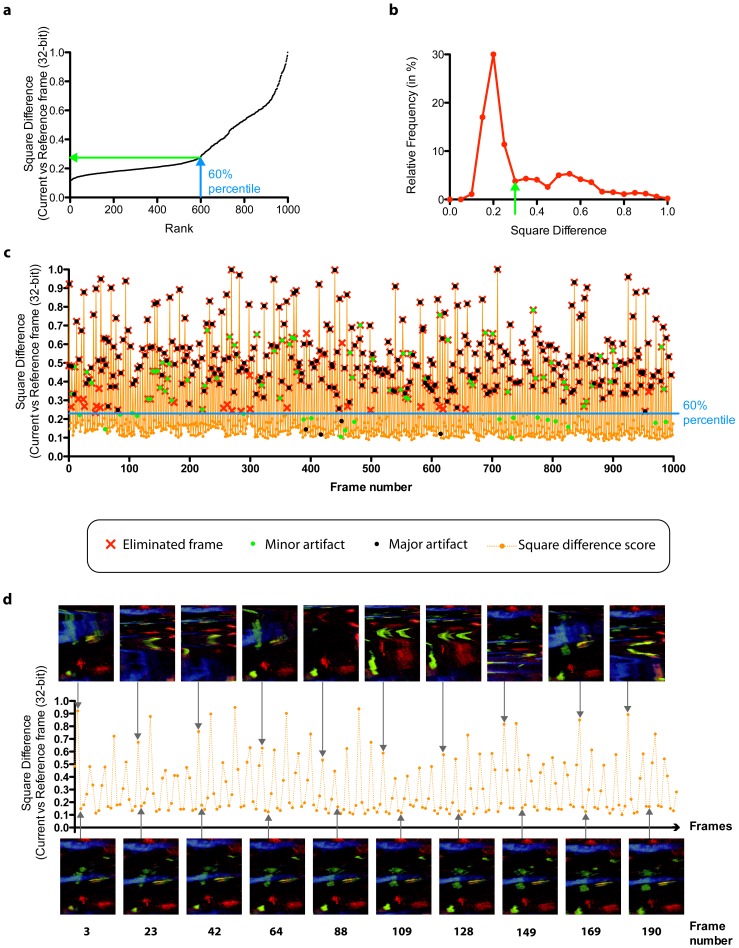
Calculation of dissimilarity scores using the automatic reference frame generation (RFG) option, and subsequent removal of artifactual frames from the video sequence. (**a**) Graph plotting of the total number of frames in a 1000-frame video against the dissimilarity score, identified as ‘square difference’ score on the y-axis. (**b**) Relative frequency distribution of the number of frames per dissimilarity score. (**c**) Graph showing the frame-by-frame analysis of a 1000-frame video sequence. Artifactual frames were eliminated (identified by red crosses) using a cutoff percentile of 60% (defined by the horizontal blue line). Major artifacts (shown as black dots) are typically artifacts resulting in a full field distortion of the image, while minor artifacts (shown as green dots) are small glitches in frames that do not impair the image interpretation. (**d**) Images corresponding to local maxima (upper panels) and minima (lower panels) in the first 200 frames of the video sequence. Note that frames corresponding to a local maximum are associated with heavy artifacts, while frames corresponding to a local minimum are typically very similar to the reference frame. All images are derived from Video S3.

It thus became interesting to reject the highest dissimilarity scores using a percentile filter. With a cutoff percentile of 60%, we were able in most cases to reject nearly 100% of the artifacts, which made the rate of artifactual images to drop from 39.8% to 2.4% in the provided example (**Figure 2** and **Video S3** and **Table S1**). It is however advantageous to compare groups of *n* neighboring images and filter out the image with the highest dissimilarity score when the percentile gating is set low, as it would otherwise result in removing too many good frames. Because the relative maximum filtering option does not allow the elimination of two consecutive distorted images, filtering the image sequence twice with the same filter (double filtering) can help remove most of the local maxima (**Figure S4**).

**Figure 3:**
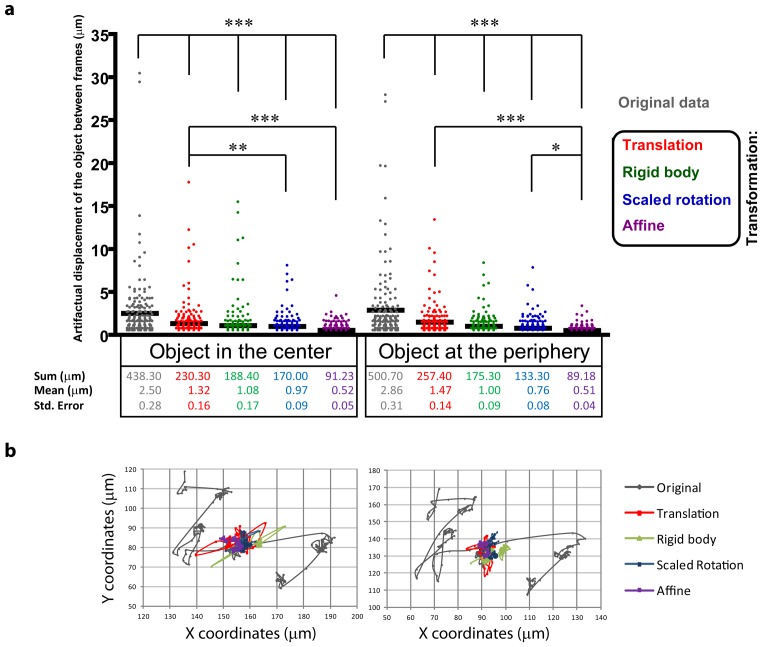
Comparison of *x–y* transformations efficiency to correct for motion artifacts induced by peristaltism in the murine myenteric plexus. (**a**) Artifactual displacement from frame to frame for an object either located in the center (object identified by the white cross in Video S5) or periphery (white circle in Video S5) of the field of view. The ‘Sum’ indicates the total artifactual displacement of an object in the video sequence. (**b**) Plots illustrating the relative motion of selected objects within the field of view. ***p<0.001, **p<0.01, and *p<0.05, one-way ANOVA with Bonferroni post-test.

Convenient extra tools were added to the program such as *x-y* alignment of the image sequence, automatic channel subtraction, time stamp and scale bars overlays. In brief, the possible alignments include: 1) *translation*; translates the source image, 2) *rigid body*; translates and rotates, 3) *scaled rotation*; translates, rotates and enlarges or reduces, and 4) *affine*; translates, rotates, shears, skews, and enlarges or reduces. The *translation* feature was particularly useful to correct for the *x–y* motion caused by breathing in brain and spinal cord videos, while the *affine* transformation was more efficient in tissues prone to peristaltism (e.g. myenteric plexus) because of the observed changes in shape, volume, and position (see **Figure 3** and **Video S5**). It should be emphasized that the *rigid body*, *scale rotation* and *affine* transformation options rely on changing pixel positions. The *translation* transformation, which is the most frequently used of all transformations, has an effect on the image as a whole, without being able to alter a separate part of the image specifically. Since the alignment proceeds by propagation from the previous frame, the *x–y* alignment efficiency was dramatically improved by the removal of artifactual frames. The channel subtraction option was implemented to remove cross-contamination of dyes in other channels, due to the simultaneous excitation of fluorophores (e.g. CFP in the YFP channel). Finally, the macro is able to display an accurate timestamp on the video, even when frames are removed.

In addition, various image-processing options were incorporated in the macro, such as maximum intensity projection and average intensity projection (**Videos S6–S7**). These options are useful to improve the signal-to-noise ratio and frame rate (frequency). We also implemented a Kalman filter that allows the removal of small artifacts within frames, such as warping and blinking when the scanning speed is too slow. Although this feature is inefficient in the presence of major artifacts and fast moving objects, it becomes extremely useful following artifact removal by the macro because it removes the noise and enhances small details. Since this filter improves the image quality by using previous frames as templates for calculation, it keeps the current video frame rate, which is a major benefit compared to a maximum intensity projection or an average intensity projection. As shown in **Videos S8, S9, S10, S11, S12**, this filtering option was essential for imaging subtle movements of microglial processes and morphological details of neurites.

## Discussion

To summarize, we report here the creation of a new ImageJ macro program for automated removal of frames contaminated with movements artifacts caused by cardiac and respiratory cycles and muscular and vascular tone during *in vivo* imaging studies. The “Intravital_Microscopy_Toolbox” program has been extensively tested and proven effective in solving problems related to tissue movements in series of images acquired from the brain, spinal cord, sciatic nerve and intestine (myenteric plexus) in living mice. Compared with manual processing of data, the automatic processing conferred by the program not only save time and effort, but also reduces the risks of errors and biases introduced by the experimenter. In addition, several key features were incorporated in the program such as filtering options, extended field of view with maximum or average projections, image-to-image alignment, channel subtraction, and automatic time stamp and scale bar overlay. The “Intravital_Microscopy_Toolbox” ImageJ plug-in, its User’s Guide and high resolution Videos S1, S2, S3, S4, S5, S6, S7, S8, S9, S10, S11, S12 have been made freely available to the public and can be downloaded from the (Toolbox S1 & Users Guide S1) or at the following websites: http://stevelacroix.crchuq.ca and http://denissoulet.crchuq.ca.

Image distortion caused by movements such as breathing is a major impediment to creating movies out of image sequences captured by 2P-IVM. If not filtered and removed from the image sequence, shifted and distorted frames will alter the quality of any time-lapse movie. Although strategies were developed to circumvent problems related to intrinsic animal movement, such as animal intubation [7], [8], [18], cardiopulmonary bypass [9], induction of muscle paralysis [11], and deep anesthesia using powerful anesthetic cocktails [10], we feel that none of these strategies are perfectly compatible with the long-term goal of repeatedly imaging the same animal. Endotracheal intubation, for example, has been used in the setting of mechanical ventilation under general anesthesia to regularize breathing and allow for steady-state conditions during which *in vivo* imaging can be triggered [7], [8], [18]. However, like cardiopulmonary bypass, this technique is a complicated and invasive surgical procedure in small laboratory rodents. The use of injectable anesthetic agents combined with muscular relaxants, such as the ketamine-xylazine-acepromazine anesthetic cocktail, was recently shown to be useful for minimizing movement during 2P-IVM, as it not only induces deep anesthesia but also reduces heart rhythm [10]. However, because these anesthetic agents are often associated with heart arrhythmias, low blood pressure, and respiratory depression, overdoses are common with these drugs and often fatal. Repeated injections will therefore multiply the chances of the animals dying from an overdose, which is a major limitation when one has to deal with costly and rare animals on which were performed complicated surgeries. In this paper, we present a new and simple post-processing method that controls for intrinsic motion artifacts without the need to perform invasive surgeries or administrate drugs that may interfere with physiological properties of the tissue.

The Intravital_Microscopy_Toolbox program presents a broad range of advantages. What perhaps distinguishes it most from other existing programs is that it is available on the free, multi-platform and widely used image analysis software ImageJ, meaning that it has the potential to be used on any intravital microscopy video recordings. The Intravital_Microscopy_Toolbox thus offers an easy and reliable filtering method for laboratories specialized in *in vivo* microscopy without the need to buy costly external devices. The removal of motion artifacts is accurate, rapid and fully automated, and therefore not subjected to errors and biases that could be introduced by the operator. On a more practical scale, the removal of distorted and out-of-focus frames allows a better understanding of biological processes and phenomena occurring within tissue of living animals. Along the same line, the complementary features of the Intravital_Microscopy_Toolbox, such as projections, channel subtraction and transformations, increase the quality of videos and facilitate data interpretation and analysis. Additionally, the program allows to efficiently exploit plugins (e.g. StackReg, JavaSIFT) that were initially developed to realign and/or transform series of images obtained from fixed-tissue sections or using *in vivo* methods that are less prone to motion artifact than 2P-IVM, such as positron emission tomography and functional magnetic resonance imaging [19], [20]. Without first filtering image sequences from 2P-IVM-generated movies to remove artifactual frames, we found that the above-mentioned plugins almost always create erroneous alignments.

It should be noted that at least one other study has developed an ImageJ-based macro (implemented in the UnwarpJ plugin) to correct motion artifacts in time-lapse videos [21]. The methodology was found efficient in resolving single cell movement relative to global tissue distortion using data sets obtained from slice cultures. However, we found that the unwarping method is inefficient when the distortion is not uniform, as it is almost always the case in tissue prone to a high the degree of movement, such as the living spinal cord, sciatic nerve or intestine myenteric plexus. The same limitations are likely to apply as well to the Matlab-based software recently developed by Greenberg and Kerr [22]. Although we did not test this program yet because it is not available in the public domain, the authors themselves state that their program is time-consuming and specifically designed for correction of movements of limited amplitude (head-fixed rats). It should be pointed out that the later methodology has the distinct advantage of not requiring the collection of a reference frame or channel. An additional drawback of the above two mentioned programs is that they rely on local changes of pixel positions within the image, a manipulation that is incompatible with publication guidelines of many journals. Importantly, the “Intravital_Microscopy_Toolbox” allows to process images as a whole rather than in parts, except when making use of the *rigid body*, *scaled rotation*, and *affine* options to rotate images, enlarge or reduce image size and/or transform images afflicted by shears and skews.

More recently, an ingenious and effective technology designed specifically for live animal imaging was introduced by Laffray et al. [12]. The technology consists of an optical stabilization sensor, fully compatible with 2P-IVM, that monitors the animal position to refocus the objective onto the plane of interest dynamically. The main disadvantages of this movement compensation device are its cost and the fact that it has yet to be implemented on a commercial system. In contrast, the computer-assisted motion filtering algorithm presented in this paper is free and compatible with both commercial and custom-built multiphoton microscopes. This was made possible by the implementation in the macro of a module that loads any RGB 24-bit image sequences, thus ensuring full compatibility with other systems.

As with any other programs, the “Intravital_Microscopy_Toolbox” also has some limitations, which gives place to future improvements. Perhaps the main limitation of the macro is that it does not allow the generation of more good frames in a video sequence, much like any other post-processing methods, but rather focuses on eliminating bad ones. Thus, the program requires that only a subset of frames are affected by distortions, otherwise too many would be thrown out. Our experience has been that this limitation becomes much less noticeable with accelerated image acquisition, as this increases the good-to-bad frame ratio. If the scan speed is set to slow (i.e. less than a couple of frames per second), fast moving objects, such as circulating cells in blood vessels, will appear as lines in images and the filtration will be impaired (with or without projections or Kalman filter). In order to work to its full potential, the macro requires a reference signal (structure) not subjected to plasticity under conditions of stress or injury for proper alignment of the frames. In the present study, we chose blood vessels as a reference structure. One needs to keep in mind, however, that blood vessels have been shown to grow or regress in response to factors released during the tissue repair process and during various pathological conditions such as cancer and neurological disorders [23], [24]. However, this phenomenon usually occurs over a protracted period of time and is not a major problem for short imaging sessions (2–3 h). The macro is also unable to process more than three fluorescent channels at the same time since the macro is designed to split RGB images in 3 single channels. Finally, the Intravital_Microscopy_Toolbox has not been conceived for 4D movies. However, we are currently working on a way to support a fourth channel and implement the processing of 4D movies with assignment of pseudo colors to channels in a 2.0 version that we intend to make freely available to the user as an update of the program.

## Acknowledgments

We acknowledge P. Thévenaz, U.E. Ruttimann and M. Unser (École Polytechnique Fédérale de Lausanne, Switzerland) for the use of their StackReg and TurboReg plug-ins as scripts that are called from the macro to perform the *x–y* realignment. We also acknowledge Stephan Saalfeld and Christopher Philip Mauer for the use of the JAVASIFT and Kalman stack filter plug-ins, respectively. Finally, we acknowledge Tiago Ferreira for the modification of his distribution plot code in the macro. We thank Nadia Fortin and Nicolas Vallières for their invaluable technical assistance. We are grateful to Éric Lebel (Olympus Canada Inc.) for providing advices on the configuration of the two-photon system. J.C. is a trainee from École Nationale Supérieure de physique de Strasbourg (Télécom Physique Strasbourg), France.

## Supporting Information

Figure S1 **Schematic representations of the restraining devices used to perform two-photon intravital microscopy in the normal and injured/diseased mouse brain (a), spinal cord (b), sciatic nerve (c), and myenteric plexus (d).**
(JPG)

Figure S2 **Impact of the choice of the single reference frame and reference channel on the dissimilarity scores in the image sequence.** For the reference channel, the Red (Texas Red-labeled blood vessels) and Blue (Thy1-CFP-labeled axons) channels were compared. Images analyzed are derived from Video S1 (right panel).
(JPG)

Figure S3 **Calculation of dissimilarity scores using a single reference frame in a short video (<200 frames).** Note that frames corresponding to a local maximum for dissimilarity scores are associated with heavy artifacts, while frames corresponding to a local minimum are typically very similar to the reference frame. Images are derived from Video S1 (right panel) and frame #079 was used as a reference.
(JPG)

Figure S4 **Comparison of the single versus double filtering process using the 3-, 5- or 7-neighbors filters to remove local maxima.** (**a**) Single filtering using a single reference frame to calculate the dissimilarity scores. Gaps in the black lines indicate frames that were removed from the original data (represented by the green lines in the graphs). (**b**) After the second filtering process, secondary peaks of local maxima are efficiently removed. Gaps in the orange lines indicate frames that were removed from the single filtering dataset (represented by the black lines). A single reference frame (#079) was used to calculate the dissimilarity scores using images derived from Video S1 (right panel).
(JPG)

Figure S5 **Comparison of single versus automatic reference frame generation (RFG) processing to calculate dissimilarity scores.** In long videos (>1,000 frames), the baseline of the dissimilarity scores is not linear when a single reference frame is used. The use of the automatic RFG option allows to keep the baseline profile as linear as possible, which increases the amplitude of local maxima and allows better removal of artifacts. Note that minor artifacts are too small to alter significantly the interpretation of the videos, yet they should still be considered as artifacts because they affect the visual quality of the videos.
(JPG)

Table S1 **Percentage of remaining artifactual images following correction of movement artifacts using the various filtering options available in the Intravital_Microscopy_Toolbox Macro.** Images are derived from a 1000-frame video sequence (video S4) taken from the living mouse sciatic nerve.
(DOC)

Video S1 **Typical movement artifacts observed by intravital microscopy in the living mouse spinal cord, myenteric plexus, and sciatic nerve.**
(M4V)

Video S2 **Movement artifacts are detected in the brain (cortex, left panel) and spinal cord (right panel) even after implantation of a chronic cranial/spinal cord window.**
(M4V)

Video S3 **Filtering for movement artifacts in the mouse sciatic nerve.** Comparison of the original version of the video (left panel) with the ones processed using a single reference frame with a 2×3-neighbors filter (middle) or an automatic reference frame generation (RFG) with a 2×3-neighbors filter (right).
(M4V)

Video S4 **Filtering for movement artifacts in the mouse spinal cord using the automated reference frame generation (RFG) method with a cutoff percentile of 75%.**
(M4V)

Video S5 **Filtering for movement artifacts in the mouse intestine using the** ***Affine*** **transformation option implemented into the Intravital_Microscopy_Toolbox.** *X-Y* alignment of the image sequence taken from the myenteric plexus using the *Affine* transformation allows to get rid of the drift problems that occur in tissue prone to peristaltism. The white cross and circle are pointing to cells in the center and periphery of the field of view, respectively, that move in the *x-y*-direction in the original video (left panel) because of the intrinsic movement of the living intestine. Note that this movement is corrected after applying the *Affine* transformation (right panel).
(M4V)

Video S6 **Extended field of view using the maximum intensity projection option available in the macro.** Here, the original video (upper left panel) is compared with videos subjected to double filtering using the 3-neighbors filter (upper right), maximum intensity projection without filtering (lower left), and maximum intensity projection after double filtering using the 3-neighbors filter (lower right). Note in the lower left panel the increased appearance of motion artifacts when maximum intensity projection is performed without prior removal of artifacts.
(M4V)

Video S7 **Improvement of the signal-to-noise ratio using the average intensity projection option.** The original video (left panel) is compared with videos subjected to average intensity projection (middle panel), and maximum intensity projection (right panel). Before using these post-processing options, image sequences obtained from the living mouse spinal cord were filtered to remove artifactual frames using the automatic RFG method with a cutoff percentile of 55%. As illustrated by this video, the average intensity projection option is particularly useful when the noise is predominant in the video.
(M4V)

Video S8 **Filtering for movement artifacts using the Intravital_Microscopy_Toolbox improves our ability to visualize fine movements of microglial processes in the mouse brain cortex.** The original video (left panel) is compared with videos processed by either Kalman filtering (center panel) or Kalman filtering and *x–y* transformation using JavaSIFT (right panel). The dashed-line circle marks a blood vessel to better visualize the movement of the object during the imaging period. Microglial cells were pseudo-colored in white based on GFP expression in the Thy1-eCFP; CX3CR1-eGFP transgenic mouse.
(M4V)

Video S9 **Filtering for movement artifacts and improvement of the signal-to-noise ratio of an** ***in vivo*** **time-lapse video of the mouse lumbar spinal cord taken through a spinal cord window preparation.** The original video (left panel) is compared with a video processed by Kalman filtering (right panel). As illustrated here, the Kalman filter option is particularly useful when the noise is predominant in the video, and when objects don’t move too fast from frame to frame. Using the Kalman filter in movies showing fast moving objects may generate motion blur artifacts. The chronic spinal cord window chamber was implemented based on the method of Farrar et al. (2012).
(M4V)

Video S10 **Filtering for movement artifacts and improvement of the signal-to-noise ratio of an** ***in vivo*** **time-lapse video of the mouse lumbar spinal cord taken through a spinal cord window preparation.** The original video (left panel) is compared with a video processed by Kalman filtering (right panel). The chronic spinal cord window chamber was implemented based on the method of Farrar et al. (2012).
(M4V)

Video S11 **Filtering for movement artifacts and improvement of the signal-to-noise ratio of an** ***in vivo*** **time-lapse video of the mouse brain taken through a skull window preparation.** The original video (left panel) is compared with a video processed by Kalman filtering (right panel).
(M4V)

Video S12 **Filtering for movement artifacts and improvement of the signal-to-noise ratio of an** ***in vivo*** **time-lapse video (acquired at 15 fps) of the mouse brain taken through a skull window preparation.** The original video (left panel) is compared with videos subjected to double filtering using the 3-neighbors filter (middle) or double filtering using the 3-neighbors filter followed by a Kalman filtering (right panel). Note in the right panel that the filtering process removes very subtle motion artifacts and that the noise is strongly reduced using the Kalman filter.
(M4V)

Users Guide S1 **Users Guide_IVM Toolbox v1.0.pdf.** The User’s Guide document contains all the information about the installation and utilization of the Intravital_Microscopy_Toolbox v1.0 program. The guide also includes an exhaustive description of all the options described in this article.
(PDF)

Toolbox S1 **Intravital_Microscopy_Toolbox v1.0.ijm.** The Intravital_Microscopy_Toolbox v1.0 is the program that filters images. Its installation and utilization are described in the Users Guide_IVM Toolbox v1.0.pdf.
(IJM)
