# Supplementary figures and images for: Automated Filtering of Intrinsic Movement Artifacts during Two-Photon Intravital Microscopy
Source: PLoS One. 2013 Jan 11;8(1):e53942. doi: 10.1371/journal.pone.0053942 (PMC3543396; doi:10.1371/journal.pone.0053942)

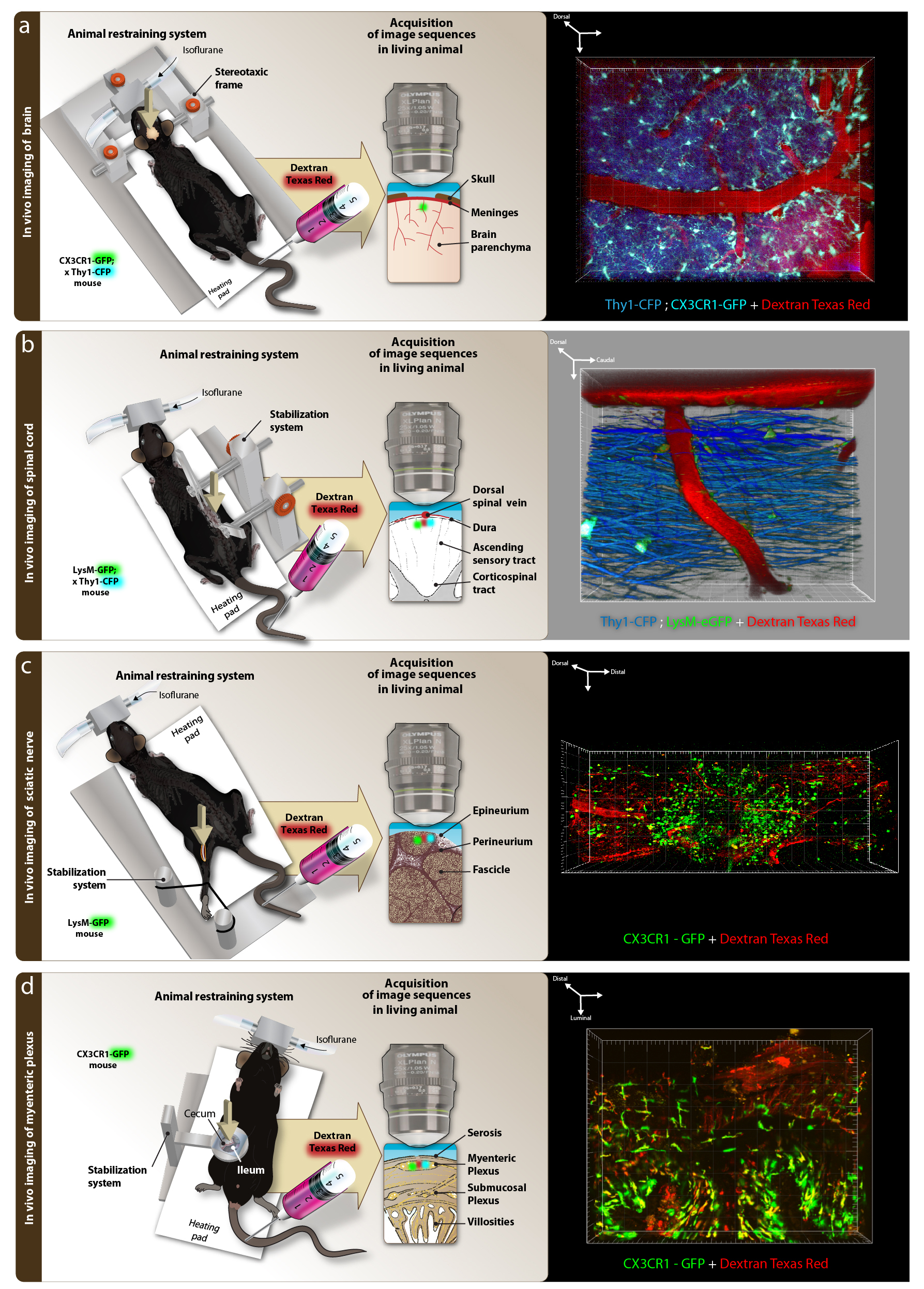

Supplement: Figure S1 — Schematic representations of the restraining devices used to perform two-photon intravital microscopy in the normal and injured/diseased mouse brain (a), spinal cord (b), sciatic nerve (c), and myenteric plexus (d). (JPG) [file pone.0053942.s001.jpg]

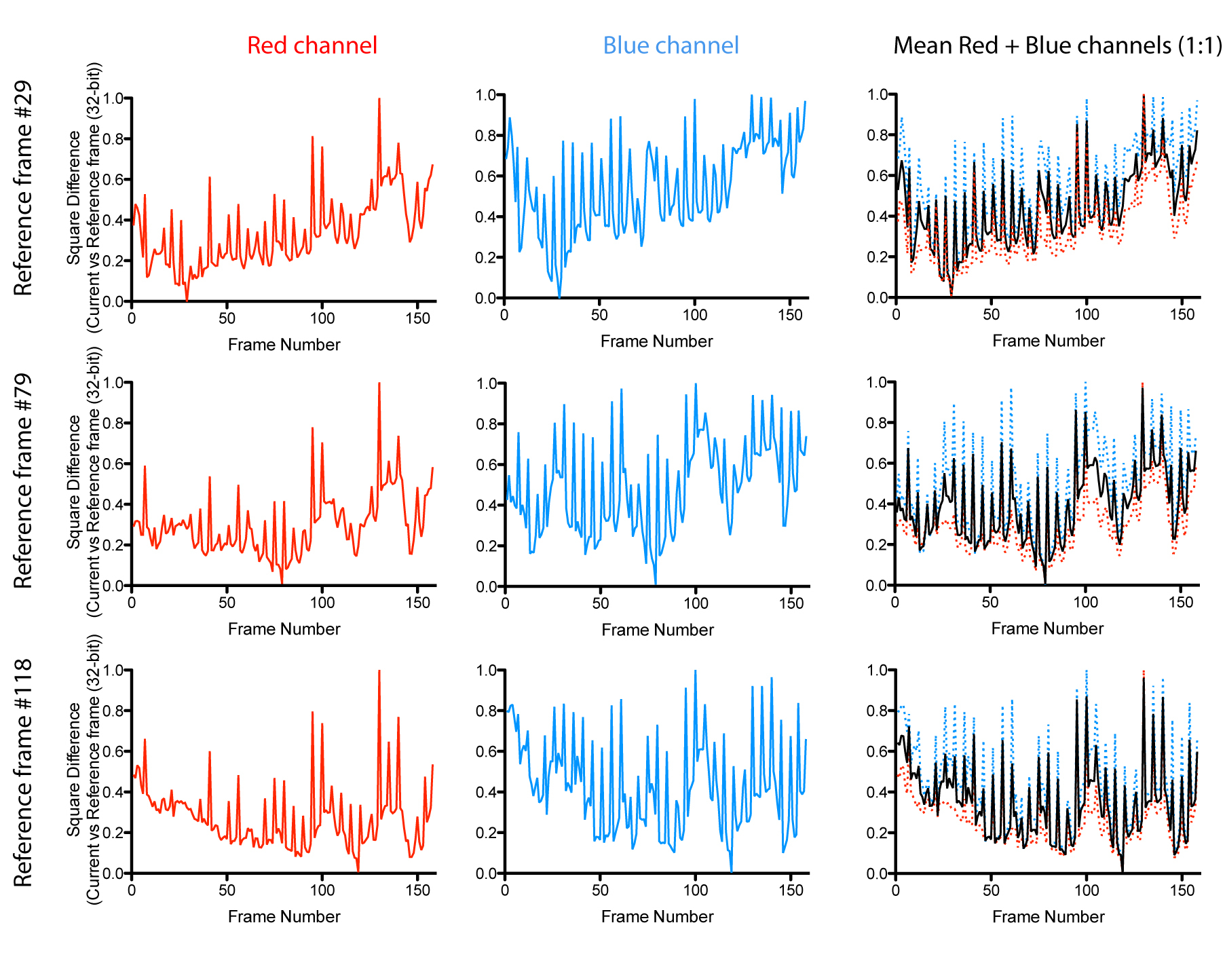

Supplement: Figure S2 — Impact of the choice of the single reference frame and reference channel on the dissimilarity scores in the image sequence. For the reference channel, the Red (Texas Red-labeled blood vessels) and Blue (Thy1-CFP-labeled axons) channels were compared. Images analyzed are derived from Video S1 (right panel). (JPG) [file pone.0053942.s002.jpg]

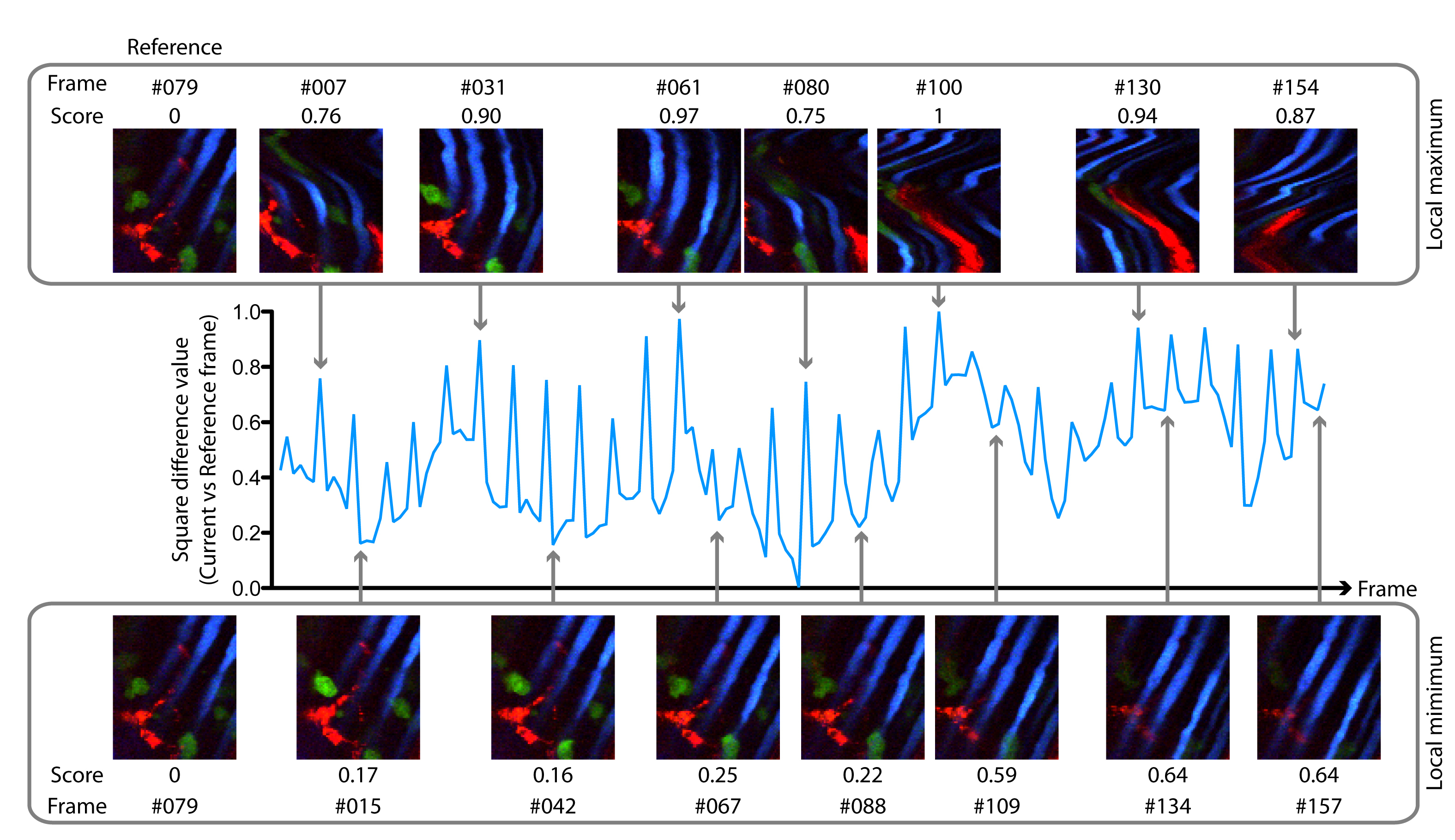

Supplement: Figure S3 — Calculation of dissimilarity scores using a single reference frame in a short video (<200 frames). Note that frames corresponding to a local maximum for dissimilarity scores are associated with heavy artifacts, while frames corresponding to a local minimum are typically very similar to the reference frame. Images are derived from Video S1 (right panel) and frame #079 was used as a reference. (JPG) [file pone.0053942.s003.jpg]

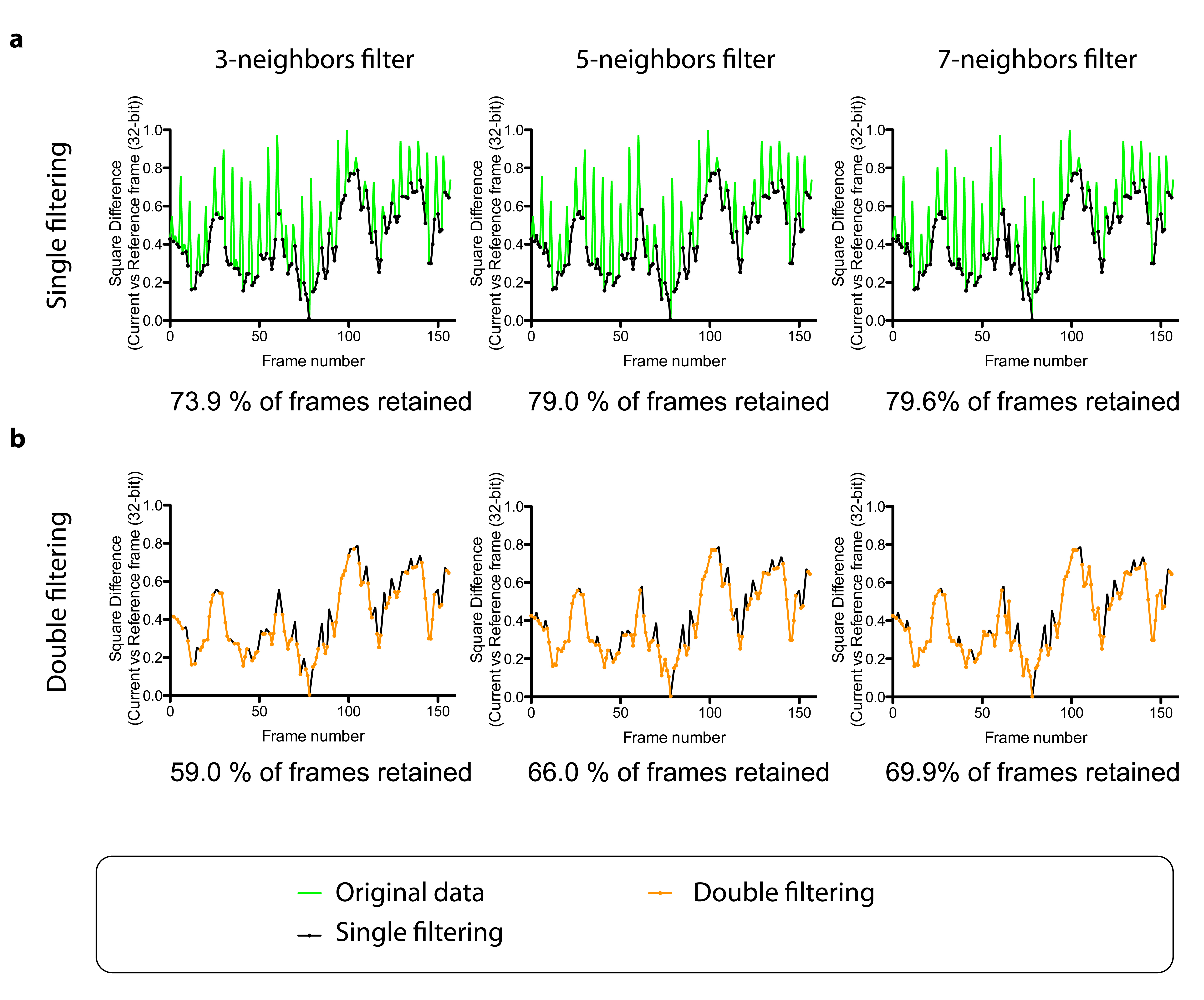

Supplement: Figure S4 — Comparison of the single versus double filtering process using the 3-, 5- or 7-neighbors filters to remove local maxima. (a) Single filtering using a single reference frame to calculate the dissimilarity scores. Gaps in the black lines indicate frames that were removed from the original data (represented by the green lines in the graphs). (b) After the second filtering process, secondary peaks of local maxima are efficiently removed. Gaps in the orange lines indicate frames that were removed from the single filtering dataset (represented by the black lines). A single reference frame (#079) was used to calculate the dissimilarity scores using images derived from Video S1 (right panel). (JPG) [file pone.0053942.s004.jpg]

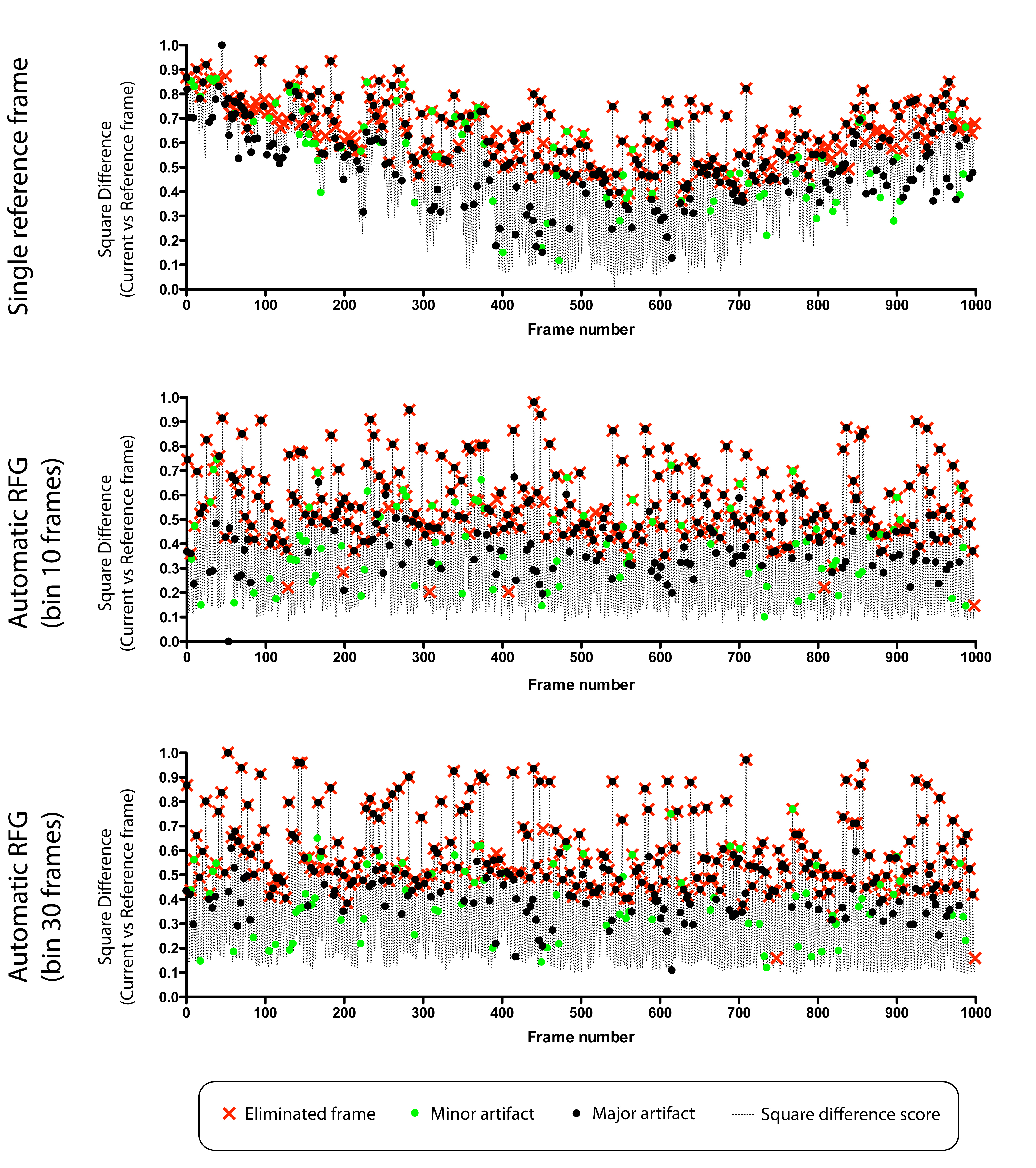

Supplement: Figure S5 — Comparison of single versus automatic reference frame generation (RFG) processing to calculate dissimilarity scores. In long videos (>1,000 frames), the baseline of the dissimilarity scores is not linear when a single reference frame is used. The use of the automatic RFG option allows to keep the baseline profile as linear as possible, which increases the amplitude of local maxima and allows better removal of artifacts. Note that minor artifacts are too small to alter significantly the interpretation of the videos, yet they should still be considered as artifacts because they affect the visual quality of the videos. (JPG) [file pone.0053942.s005.jpg]
