# Supplementary material for: Automated Filtering of Intrinsic Movement Artifacts during Two-Photon Intravital Microscopy
Source: PLoS One. 2013 Jan 11;8(1):e53942. doi: 10.1371/journal.pone.0053942 (PMC3543396; doi:10.1371/journal.pone.0053942)
Supplement: Table S1 — Percentage of remaining artifactual images following correction of movement artifacts using the various filtering options available in the Intravital_Microscopy_Toolbox Macro. Images are derived from a 1000-frame video sequence (video S4) taken from the living mouse sciatic nerve. (DOC) [file pone.0053942.s006.doc]

TABLE 1. Percentage of remaining artifactual images following correction of movement artifacts using the various filtering options available in the Intravital_Microscopy_Toolbox Macro. Images are derived from a 1000-frame video sequence (Supplementary video 4) taken from the living mouse sciatic nerve.

| **TOTAL ARTIFACTS** | Original data | Macro Automatic RFP (1000 ref. frames) **Cut-off 60%** | Macro Automatic RFP (1000 ref. frames) **3 neighbors filter** | Macro Automatic RFP (1000 ref. frames) **5 neighbors filter** | Macro Automatic RFP (1000 ref. frames) **7 neighbors filter** | Macro Automatic RFP (1000 ref. frames) **9 neighbors filter** |
| --- | --- | --- | --- | --- | --- | --- |
| Detected artifacts | 398 | 374 | 240 | 238 | 222 | 90 |
| Remaining artifacts | 398 | 24 | 158 | 160 | 176 | 308 |
| Rate of artifactual images | **39.80** | **2.4** | **15.8** | **16** | **17.6** | **30.8** |
| Specific detection rate | n.d. | 93.97 | 60.30 | 59.80 | 55.78 | 22.61 |
| False positive rate | n.d. | 6.5 | 19.73 | 1.65 | 1.33 | 1.10 |
| False negative rate | n.d. | 6.03 | 39.70 | 40.20 | 44.22 | 77.39 |
|  |  |  |  |  |  |  |
| **TOTAL ARTIFACTS** | Original data | Macro Automatic RFP (1000 ref. frames) **Cut-off 60%** | Macro Automatic RFP (1000 ref. frames) **2x3 neighbors filter** | Macro Automatic RFP (1000 ref. frames) **2x5 neighbors filter** | Macro Automatic RFP (1000 ref. frames) **2x7 neighbors filter** | Macro Automatic RFP (1000 ref. frames) **2x9 neighbors filter** |
| Detected artifacts | 398 | 374 | 106 | 88 | 46 | 47 |
| Remaining artifacts | 398 | 24 | 292 | 310 | 352 | 351 |
| Rate of artifactual images | **39.80** | **2.4** | **29.2** | **31** | **35.2** | **35.1** |
| Specific detection rate | n.d. | 93.97 | 26.63 | 22.11 | 11.56 | 11.81 |
| False positive rate | n.d. | 6.5 | 56.02 | 58.29 | 50 | 52.52 |
| False negative rate | n.d. | 6.03 | 73.37 | 77.89 | 88.44 | 88.19 |
|  |  |  |  |  |  |  |
| **TOTAL ARTIFACTS** | Original data | Macro Automatic RFP (1000 ref. frames) **Cut-off 60%** | Macro Single ref. frame **2x3 neighbors filter** | Macro Bin 5 frames (200 ref. frames) **2x3 neighbors filter** | Macro Bin 10 frames (100 ref. frames) **2x3 neighbors filter** | Macro Bin 30 frames (33 ref. frames) **2x3 neighbors filter** |
| Detected artifacts | 398 | 374 | 192 | 234 | 237 | 237 |
| Remaining artifacts | 398 | 24 | 206 | 164 | 161 | 161 |
| Rate of artifactual images | **39.80** | **2.4** | **20.60** | **16.40** | **16.10** | **16.10** |
| Specific detection rate | n.d. | 93.97 | 48.24 | 58.79 | 59.55 | 59.55 |
| False positive rate | n.d. | 6.5 | 25.87 | 7.14 | 4.44 | 2.47 |
| False negative rate | n.d. | 6.03 | 51.76 | 41.21 | 40.45 | 40.45 |

n.d. : no data.
