# Supplementary material for: Automated Filtering of Intrinsic Movement Artifacts during Two-Photon Intravital Microscopy
Source: PLoS One. 2013 Jan 11;8(1):e53942. doi: 10.1371/journal.pone.0053942 (PMC3543396; doi:10.1371/journal.pone.0053942)
Supplement: Users Guide S1 — Users Guide_IVM Toolbox v1.0.pdf. The User’s Guide document contains all the information about the installation and utilization of the Intravital_Microscopy_Toolbox v1.0 program. The guide also includes an exhaustive description of all the options described in this article. (PDF) [file pone.0053942.s019.pdf]

Université
Laval

Intravital Microscopy Toolbox User Guide

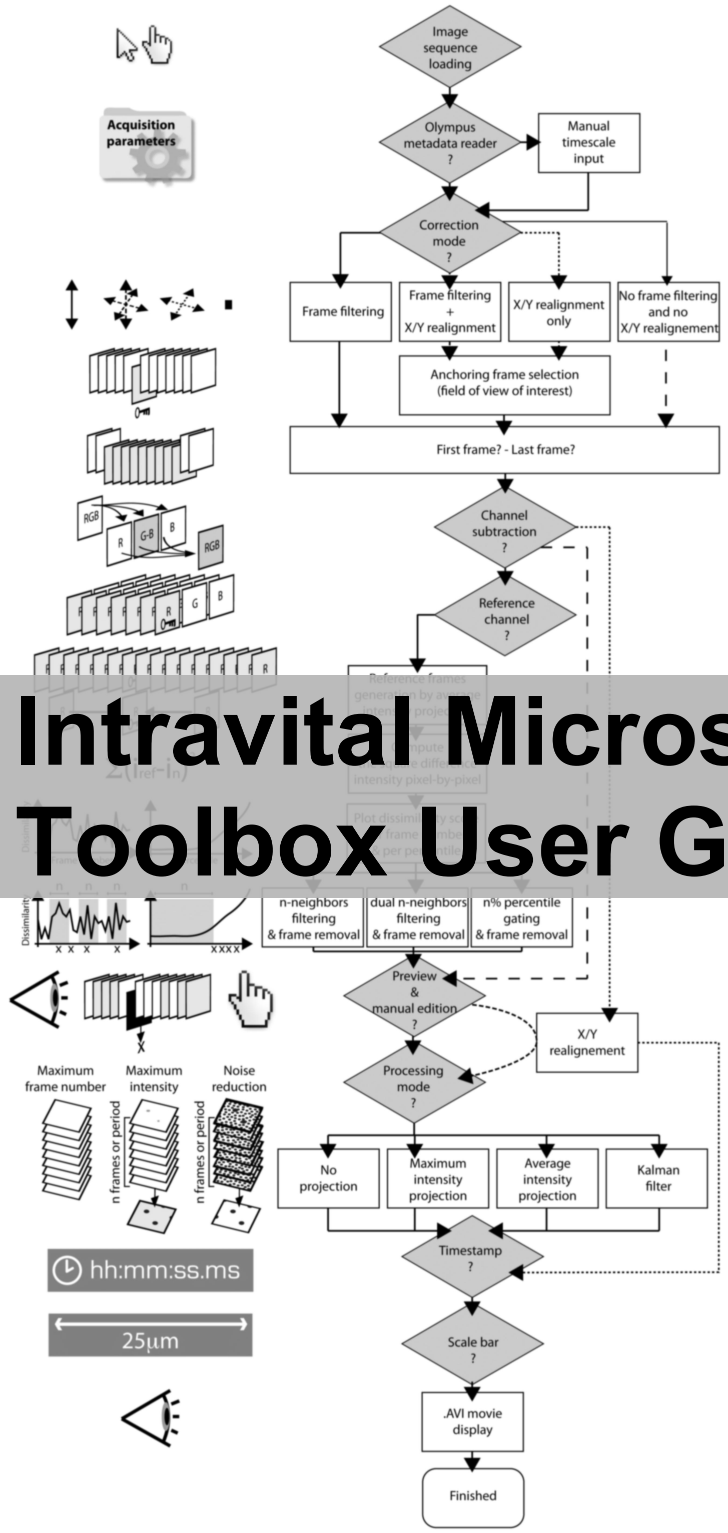

Copyright (C) 2012 Denis Soulet, Alexandre Paré, Julien Coste and Steve Lacroix

Permission to use, copy, modify, and distribute this software for any purpose without fee is hereby granted, provided that this entire notice is included in all copies of any software which is or includes a copy or modification of this software and in all copies of the supporting documentation for such software. Any for profit use of this software is expressly forbidden without first obtaining the explicit consent of the author.
THIS SOFTWARE IS BEING PROVIDED "AS IS", WITHOUT ANY EXPRESS OR IMPLIED WARRANTY. IN PARTICULAR, THE AUTHOR DOES NOT MAKE ANY REPRESENTATION OR WARRANTY OF ANY KIND CONCERNING THE MERCHANTABILITY OF THIS SOFTWARE OR ITS FITNESS FOR ANY PARTICULAR PURPOSE

If you use this macro for your research, please cite the authors:
Soulet *et al.*, PLoS One, 2012

This work has been co-developed by Dr. Denis Soulet, Alexandre Paré, Julien Coste, and Dr. Steve Lacroix all working at the CHUQ-CHUL Research Center (Neuroscience Axis) in Quebec City (QC), Canada. The authors acknowledge P. Thévenaz, U.E. Ruttimann and M. Unser (École Polytechnique Fédérale de Lausanne, Switzerland) for the use of their StackReg and TurboReg plug-ins as scripts that are called from the macro to perform the x-y realignment. We also acknowledge Stephan Saalfeld and Christopher Philip Mauer for the use of the JavaSIFT and Kalman stack filter plug-ins, respectively. Finally, we acknowledge Tiago Ferreira for the modification of his distribution plot code in the macro. We thank Nadia Fortin and Nicolas Vallières for their invaluable technical assistance. We are grateful to Éric Lebel (Olympus Canada Inc.) for providing advices on the configuration of the two-photon system. This work was supported by grants from the Parkinson Society of Canada (D.S.), Canadian Foundation for Innovation (S.L. and D.S.), Wings for Life Spinal Cord Research Foundation (S.L.), and Natural Sciences and Engineering Research Council of Canada (S.L.). A.P. is supported by a studentship from the Multiple Sclerosis Society of Canada. J.C. is a trainee from École Nationale Supérieure de physique de Strasbourg (Télécom Physique Strasbourg), France.

The authors:
Denis Soulet, PhD
Alexandre Pare, MSc
Julien Coste
Steve Lacroix, PhD

Intravital microscopy (IVM) is a powerful tool for biologists who want to visualize biological phenomena in living animals. A key aspect of this technique is the stabilization of the animal to reduce movement during the imaging session. Unfortunately, acquired images are often out of focus or distorted, in part due to cardiac and respiratory cycles. To date, the only choice scientists had was to manually remove the artifactual frames in a time consuming methods that is based on judgment only. To solve this problem, we created the *Intravital Microscopy Toolbox*, a free *ImageJ* plugin designed to automatically detect and remove artifactual frames in videos generated using IVM. Convenient extra tools were also added to the program such as *x-y* alignment of the image sequence, automatic channel subtraction, time stamp and scale bars overlays.

1. Installation.

Note: The *Intravital Microscopy Toolbox* is compatible with both the 32- and 64-bit versions of *ImageJ 1.47a* and can be runned under Microsoft Windows (7 64-bit, Vista 64-bit). Please note that the 64-bit version of ImageJ allows to allocate more memory, which can be useful to process very large videos.

1.1 Download the macro *Intravital_Microscopy_Toolbox.ijm* using the following link: http://www.stevelacroix.crchuq.ca/support-visuel_en.php
or
http://www.denissoulet.crchuq.ca/
Place the *.ijm* file in the *Plugins* folder in the *ImageJ* application file.

1.2 In order for the macro to work, you will need to download the following plugins:
   a. StackReg and TurboReg,
   b. *Kalman_Stack_Filter.class,*
   c. *imagescience.jar, Jama-1.0.2.jar* and *Java_SIFT.jar*.

The links and pathways are displayed in the **Table 1 in Appendix** (page 18 of this document). Place the folders and files in the *Plugins* folder in the *ImageJ* file. Installed plugins will be active after *ImageJ* restarts.

2. Data preparation for importation in the macro.

2.1 **Data acquisition:** it is mandatory for the videos to be saved in RGB format, i.e. each channel will be encoded in Red, Green or Blue colors, which allows a maximum of 3 channels per video. Note that the filename length cannot exceed 60 characters in total.

2.2 **Olympus users:** Since acquired images can come from various commercial microscopes (e.g. Olympus, Leica, Nikon, Zeiss), the file format can be different depending on the software system. For this reason, we developed a MetaData reader specifically for our Olympus FV1000MPE microscope to retrieve images automatically. First, it is required to export videos from the OIF format to images in TIFF format, with properties saved as ASCII file for the MetaData (use the Export File option in the Fluoview software; see **Figure 1**). When the conversion will be completed, you should see a list of images and a TXT file in the same folder of interest. For Olympus users, the TXT file can be used later to automatically retrieve acquisition parameters with the macro.

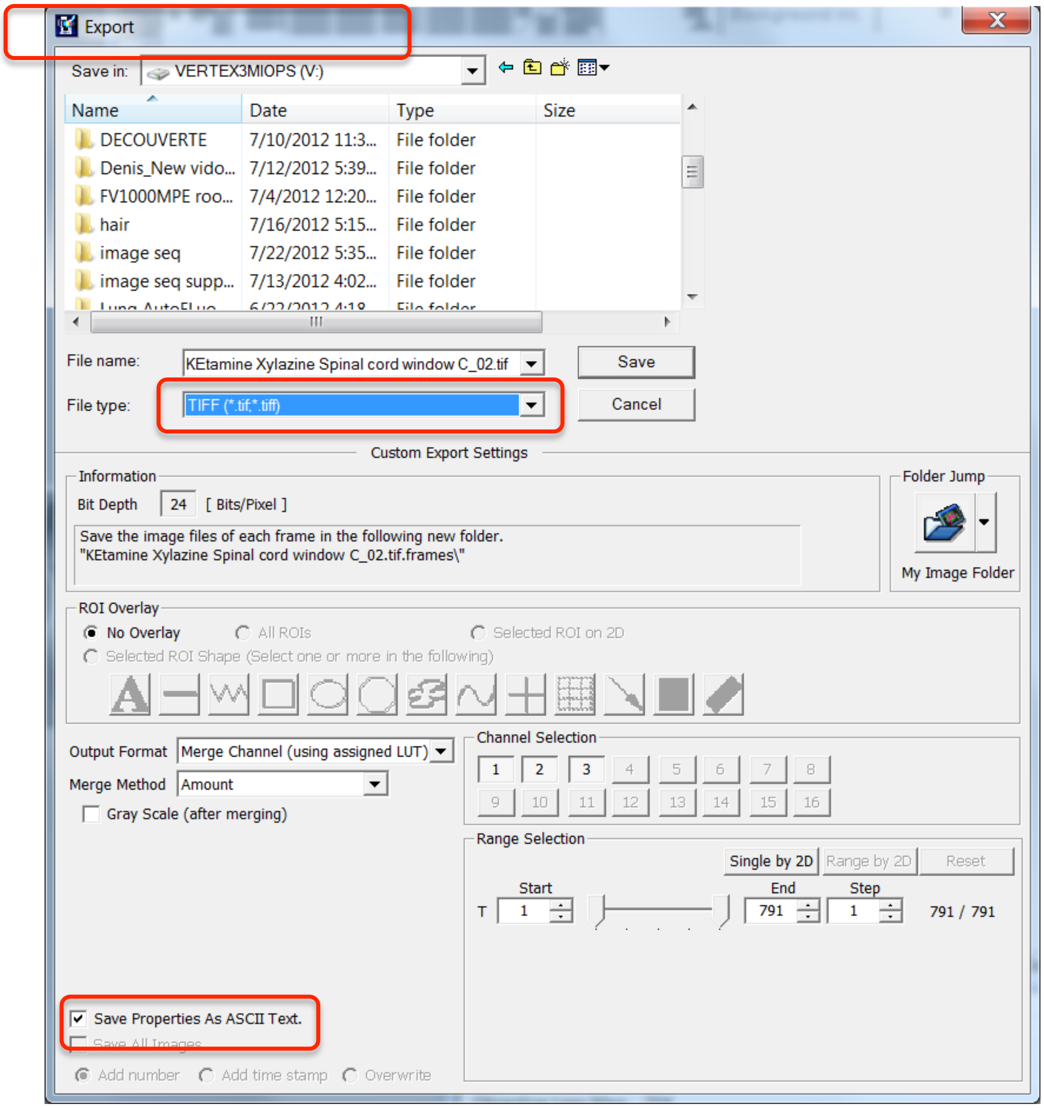

Figure 1 – Export your data

2.3 **Other users**: As an alternative for non-Olympus users, we created an optional importation module that can be used for TIFF image sequences. In that case, the video will need to be exported as a numbered RGB TIFF image series (the filename length cannot exceed 60 characters total). For this purpose, consult the manual guide of your acquisition software.

TIFF file series naming convention:
If you have TIFF images to be read into the Intravital Microscopy Toolbox, you should know about the naming convention to be used.

- Filename length < 60 characters
- Numbering starting from xxx1 and not xxx0
- Numbering including zeros, i.e. 001, 002, 003…, 010, 011, 012 …. 100

- Tiff format: RGB 24-bit

3. How to use the Intravital Microscopy Toolbox macro.

3.1 In *ImageJ*, select Plugins > Intravital_Microscopy_Toolbox.

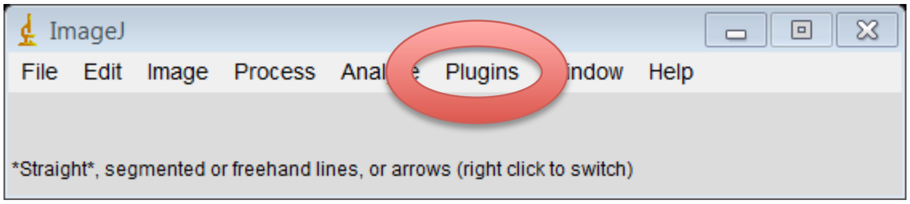

Figure 2 – Run the macro

3.2 The following window will appear:

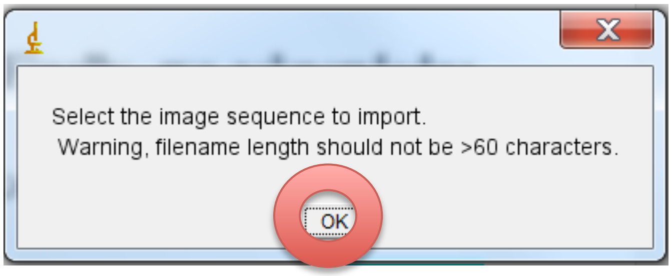

Figure 3 – Select the image sequence to import

3.3 In the new window, press OK and select the appropriate image sequence. In the next window, the number of images should be indicated with the image sequence import module in ImageJ. Make sure that the *Sort names numerically* box is checked and press OK.
Note: If you do not want to use all the images in the folder, select a different start image and increment.

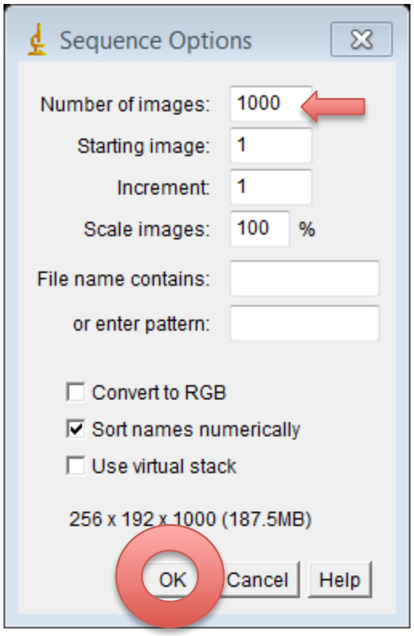

Figure 4 – Import image sequence options

3.4 In the next window, named *Choice of correction*, select between

- *Frame filtering,*
- *x/y realignment only,*
- *Frame filtering + x/y realignment*

If the imaging system you use is *Fluoview FV1000* from Olympus, all exported image sequences will include a *.txt* file that the macro can read (**2.2**). If so, check the corresponding box and jump to point **3.6**. If you use another system, uncheck the box and go to point **3.5**. Ensure the box named *Automatic reference frame generation* is checked and enter an odd number of frames (min 3) for the filter width (default is 11).

Note: Do not use the x/y realignment if the video displays a lot of artifacts, as this would result in an even more altered video. In this particular situation, we advise you to use either the *frame filtering* followed by *Kalman filter* with *SIFT correction*, or the *frame filtering* + *x/y realignment* option.

Then, press the OK button.

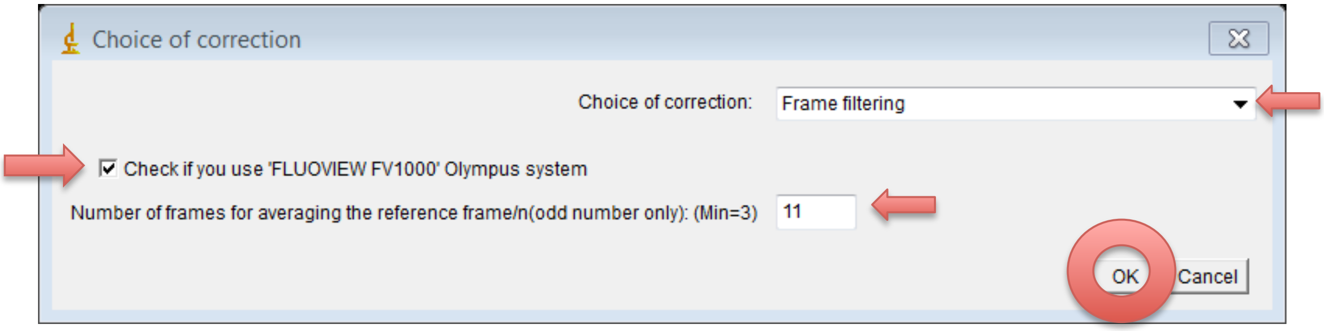

Figure 5 – Dialog box for correction mode

3.5 If you use a different system than Olympus *Fluoview FV1000*, gather the following information: time between each frame (in seconds), the time the video starts (if not 0, when for example one wants to concatenate several consecutive videos), the pixel size (in µm), and the number of digits in the file name number. **Note:** Use the dot (.) instead of the comma (,) for decimals.

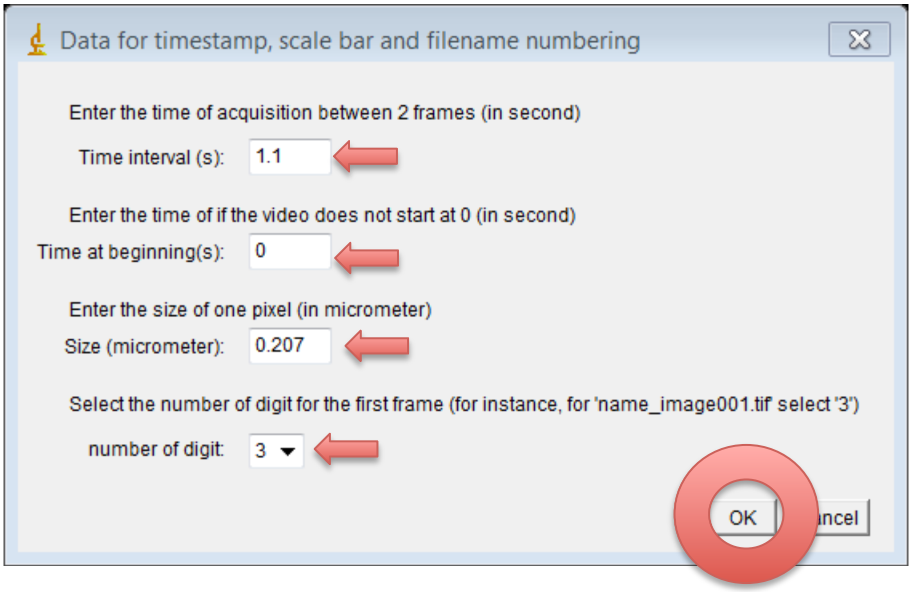

Figure 6 – Dialog box for timestamp, scale bar and filename numbering

3.6 If you choose either the *x/y realignment* or the *frame filtering* + *x/y realignment* correction (**3.4, Figure 5**), a new window will appear asking for scrolling between the images and the selection of an anchoring frame, *i.e.* a frame that is representative of the field of interest and which shows no artifact. This frame will be used as a template for the x/y realignment.

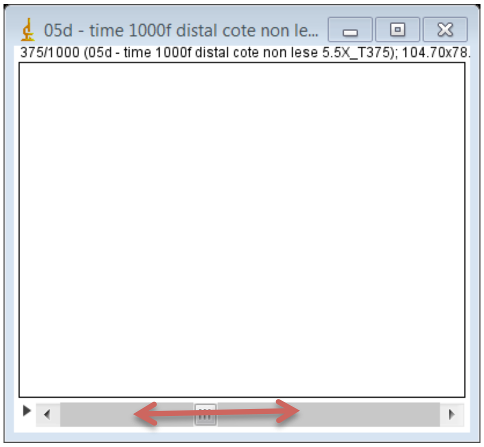

Figure 7a – Anchoring image selection

Once you have scrolled to the field of view of interest, press OK.

Note: Do not select an anchoring frame too close from the beginning or the end of the image sequence, because if this frame is removed during the filtering process, there could be no frame around to be selected

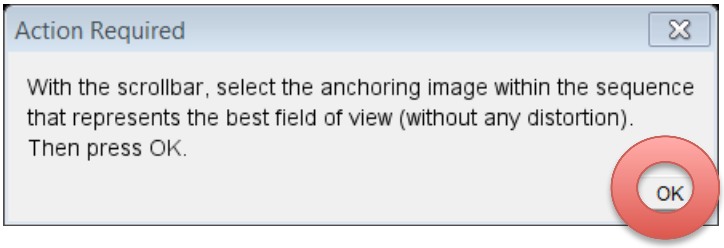

Figure 7b – Anchoring image selection

3.7 In the next window named *Methods for XY alignment*, select the transformation you want to use. For example, you can use:

- *Translation,* to correct for *x-y* motion,
- *Rigid Body,* to correct for *x-y* motion and rotation of the field of view,
- *Scaled Rotation,* to correct for *x-y* motion, rotation, enlargement and reduction,
- *Affine,* to correct for all the previous artifacts plus skewing and shearing.

For more details about the registration algorithm, please see Credits.
Then, press the OK button to continue.

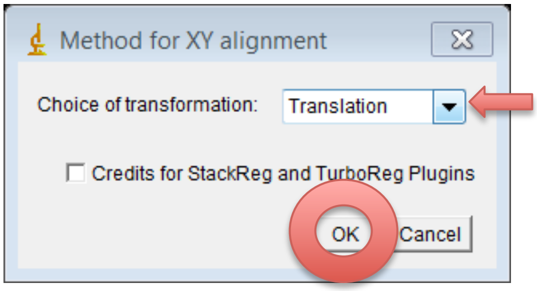

Figure 8 – XY transformation dialog box

3.8 In the next window named *Settings*, select the range of frames you want to use (start and stop). If you use the metadata reader option, then the final frame to use will be the last frame of your original video by default.
Note: If the number of the last frame is higher than the number of the last frame from the original sequence, the macro will crash. If the anchoring frame is outside the range of the first-last frame, the macro will crash.

If you cross-excite two fluorophores simultaneously (e.g. CFP and GFP, or GFP and YFP), one of the channels may leak in the other. Therefore, you will need to

subtract one channel from the other one to obtain a more specific signal. In that case, select the appropriate channel subtraction method (one check box only).

Finally, select the reference channel and press the OK button. It is critical to select only one reference channel. The generation of synthetic reference frames can take quite a while (several minutes) if the frame number and frame size is large.
Note: The signal in the reference channel should be stable during the entire sequence (e.g. blood vessels, axons, etc.).

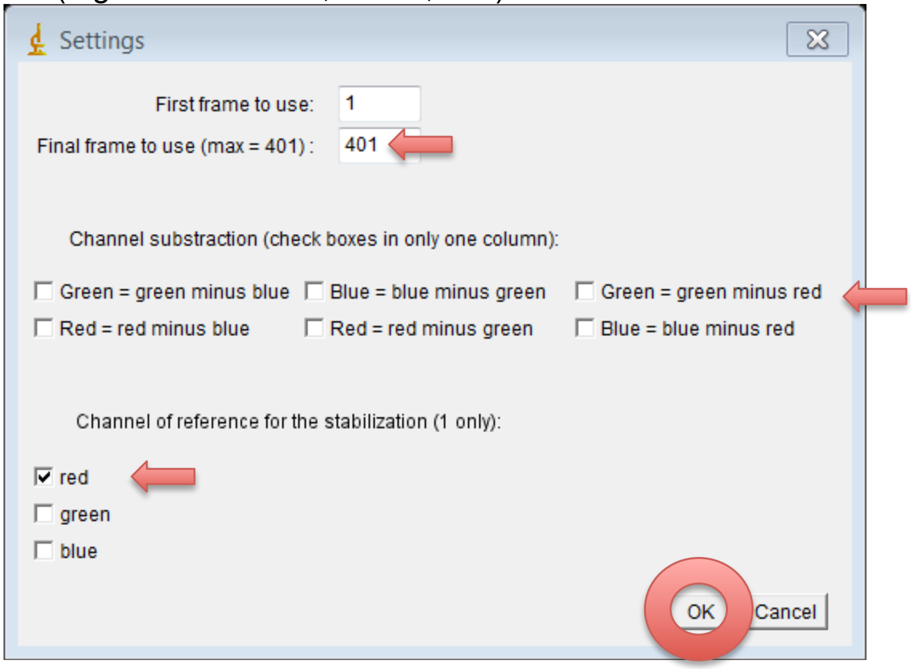

Figure 9 – Dialog box for frames processing, channel subtraction and reference channel

3.9 After a few minutes, four windows will appear:

- *Score Plot.jpeg*, (see **Fig. 10a, 10b, 11a**)
- *Square Difference vs Rank (Percentile) Plot.jpeg*, (see **Fig. 11b**)
- *Relative frequency plot.jpeg*, (See **Fig. 11c**)
- *Settings*. (See **Fig. 12**)

The first three windows are indicators of the quality (score) of the image sequence.
Note: For reference purpose, the three *.jpeg* files will be saved in the source folder, under *Results*.

In **Figure 10a, 10b and 10c**, score plots are representative examples of square difference intensity profiles (*i.e.* dissimilarity scores) with fluctuating baselines. Note that the colors of the square difference scores correspond to the reference channel that was selected for the analysis, *i.e.* red, green or blue.

Artifacts are originating either from a drift of the sample (**Fig. 10a and 10b**), or from the operator trying to compensate manually for the drift (**Fig. 10c**). The use of the maximum local filtering with n-neighbors filters is perfectly adapted to remove artifacts in this situation.

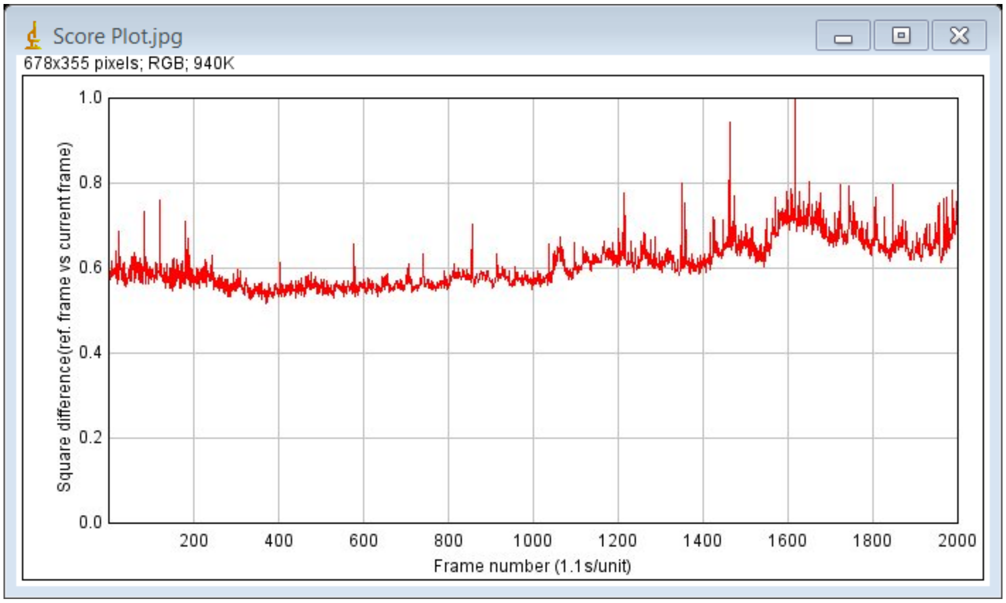

Figure 10a – Alteration in the baseline of square difference scores (automatic RFG) following a z-axis drift of the sample

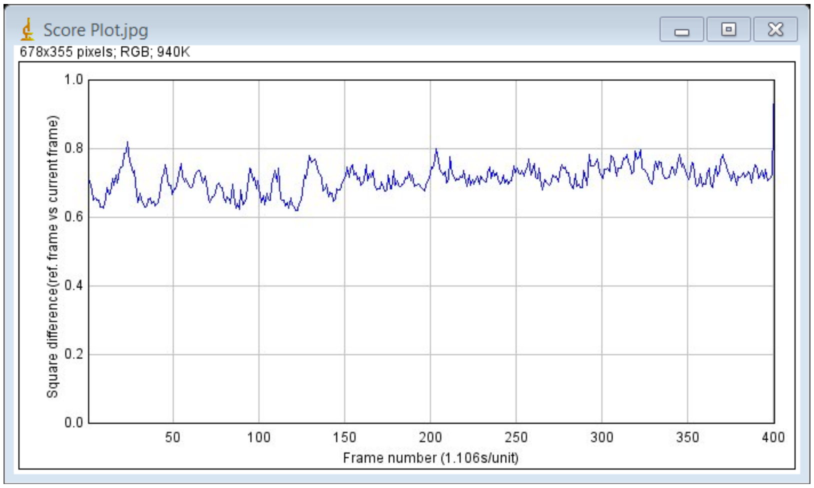

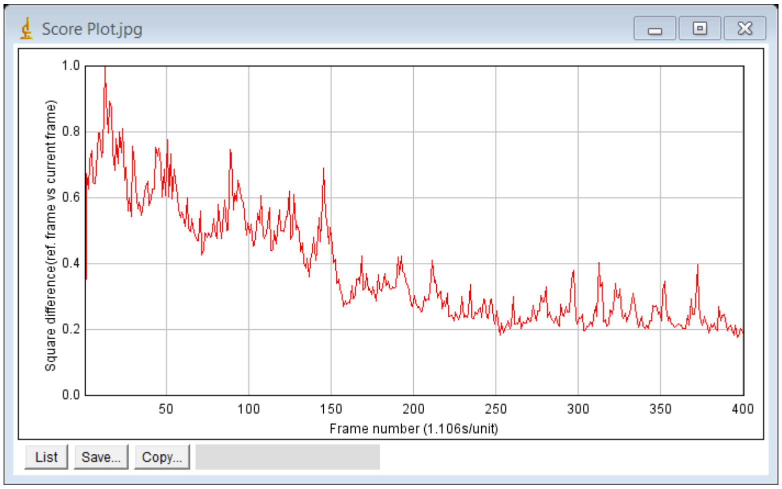

Figure 10b – Alteration in the baseline of square difference scores (automatic RFG) for the blue and red channels following a z-axis drift of the sample

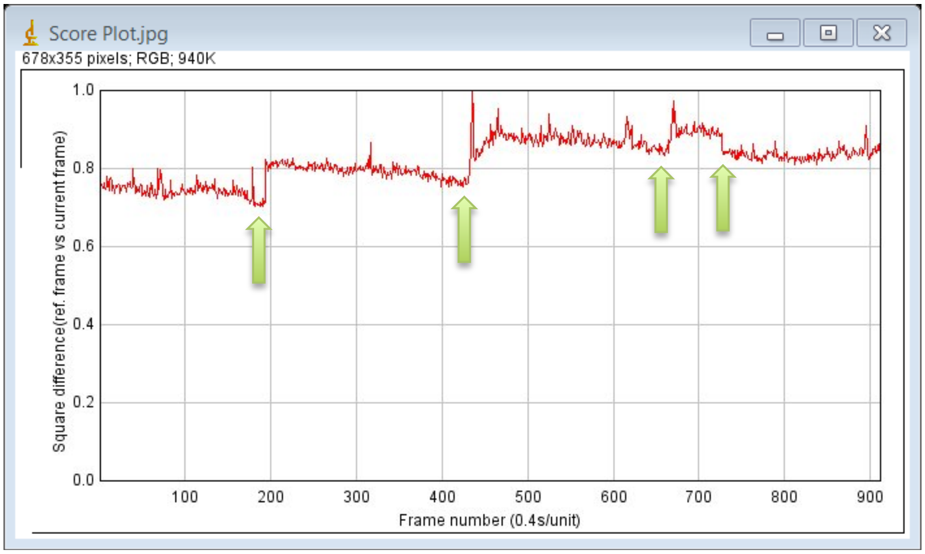

Figure 10c – The multi-plateau shape of the curve corresponding to square difference scores (automatic RFG) is normally seen when the operator has changed the z-position manually to correct for a pronounced z-axis drift during the image acquisition (Green arrows indicate a manual change of focus)

In **Figure 11a**, the score plot is a representative example of the square difference intensity profiles in the situation where the animal moves cyclically, but stays in the same range of positions, like during breathing. Since the baseline is low and relatively linear, the use of a cutoff based on percentile is particularly adapted to remove artifacts.

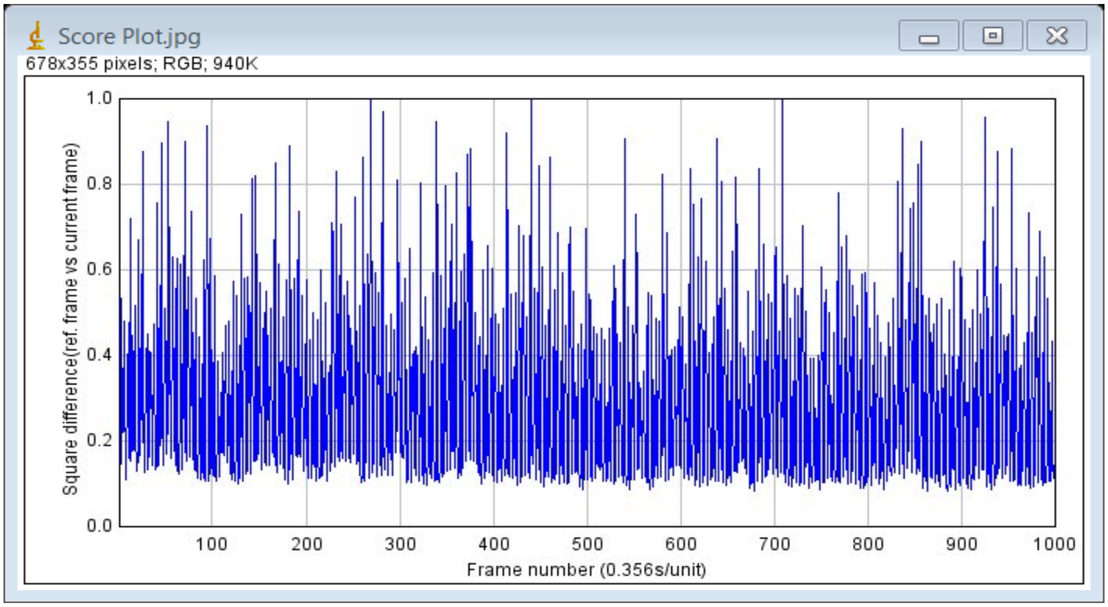

Figure 11a – Graph showing a typical example of when the z-position of the sample oscillates around a constant focal point (automatic RFG)

In this situation, when the square difference scores are plotted in function of the rank of the frames (**Figure 11b**), the dissimilarity scores tend to deviate from the extrapolation of the linear curve (green dashed line) after reaching a specific level (indicated in the plot with a blue arrow). In the provided example, when the frame rank >550 (=55% percentile), the dissimilarity scores increase dramatically, which means that 55% of the frames have very few artifacts. Thus, our suggestion would be to choose a 55% percentile value for the cutoff, to keep 55% of the frames with the lowest dissimilarity scores and reject the frames with major artifacts.

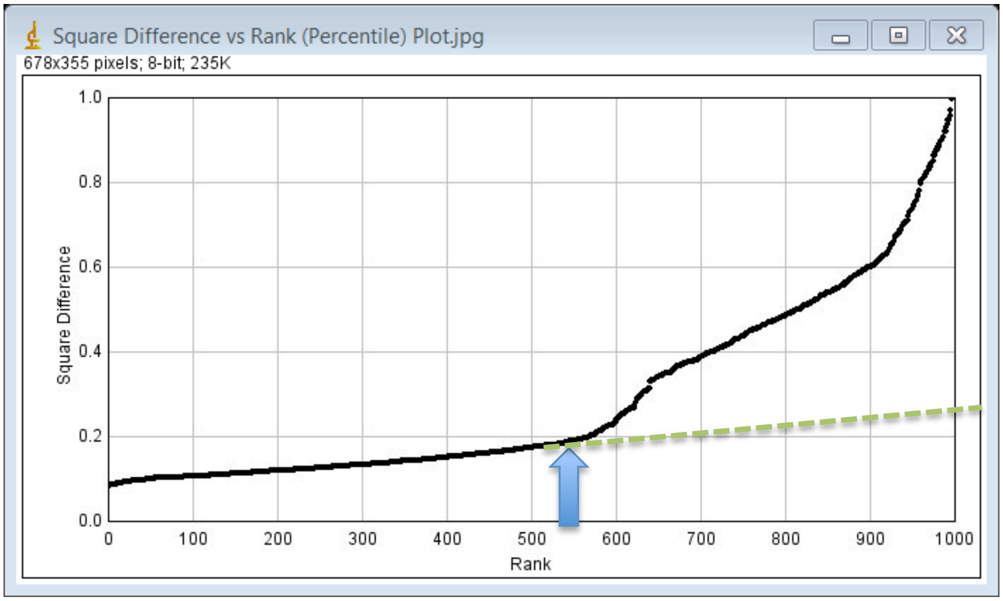

Figure 11b – Example of graphical determination of a suitable cutoff value using the score ranking. The blue arrow indicates the rank at which the dissimilarity scores begin to deviate substantially from the extrapolation of the linear part of the curve (dashed green line)

As another visual representation of the dissimilarity score profile, the relative frequency plot (**Figure 11c, red curve**) allows to see the relative quantity of frames for a specific score, which may help identify the method of choice to filter frames. Here, as indicated by the blue arrow (**Figure 11c**), if we select a square difference intensity value between 0.2 and 0.25, we will keep most of the frames belonging to the linear portion of the curve (see **Figure 11b**).

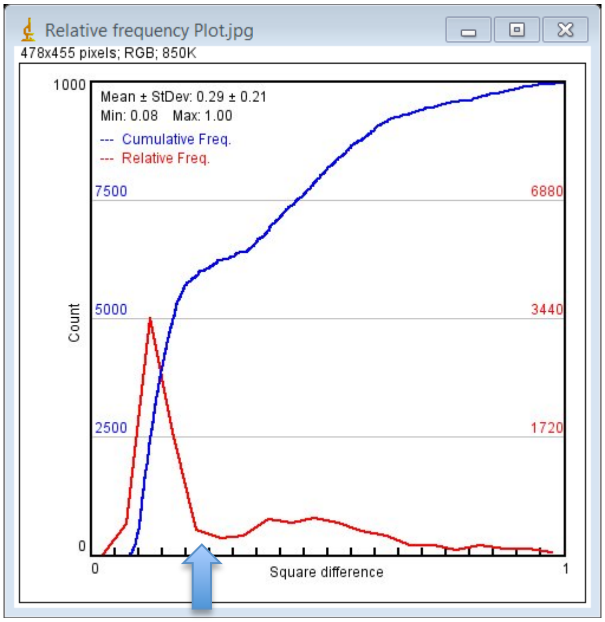

Figure 11c – Example of graphical determination of a suitable cutoff value using the relative frequency

3.10 In the *Settings* dialog box (**Figure 12**), you will next have to select a method to remove distorted frames. The choices of methods are:

- neighbor filtering (3, 5, 7, 9 and 11) or
- percentile filtering.

If you use one or several n-neighbors filter(s) (local maximum), you can run the filter twice using the *Perform second pass* option. This is useful if you expect the presence of subsequent peaks of distortion in the video.

If you select the Cutoff option, then enter a percentile value. Use the plots shown in **Figures 11a-c** to help you determine the optimal cutoff value. If you enter 40%, you will keep 40% of the frames with the lowest dissimilarity scores.
The min cutoff value is usually set to 0, but in specific conditions when the focus drops, frames become darker and the sum of square differences values can peak under the baseline. In this specific condition, one can set the min cutoff value higher to remove the dark frames in the video.

Next, select at least one type of processing (Maximum intensity, Average intensity and/or No Projection). Select whether you want to project a specific number of frames or whether you want to project frames for a specific duration of time. Then, enter the value in the corresponding box, *i.e.* a number of frames or a time in milliseconds (ms).

You can also use a Kalman Stack Filter for high gain noise reduction. In that case, enter the acquisition noise and bias values. For more details about the Kalman Stack Filter, please see the website of Christopher Philip Mauer. Since the Kalman filter reduces the high gain noise, it improves the quality of the video and allows a more efficient *x-y* alignment. For these reasons, we therefore implemented a SIFT post-Kalman filter option for better stabilization of the images (in the PC version only). For more details about the SIFT plug-in from Stephan Saalfeld, see his website.

Finally, it is possible to display the time stamp and a scale bar by checking the corresponding boxes.

Note: When the macro removes frames, it automatically adjusts the time stamp accordingly. Although frames may not be removed in a regular manner, the macro will still keep track automatically of the time between frames. This is a very useful feature of the Macro program because it allows to know precisely the time between two frames, even though the timeline becomes uneven.

Press OK when the options of interest have been selected.

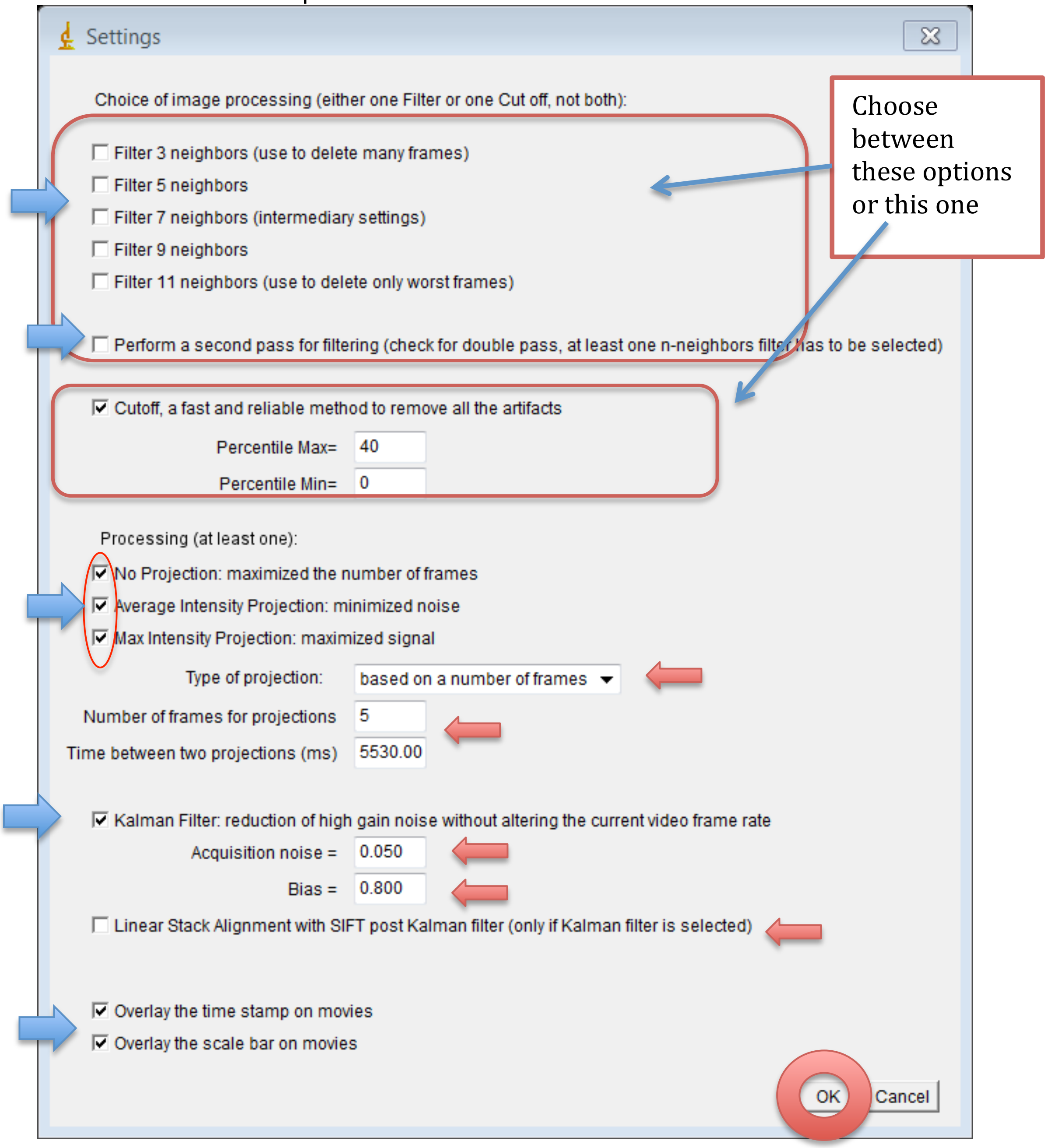

Figure 12 – Filtering settings dialog box.

3.11 After the calculations, the macro will display the results for each filtering method.

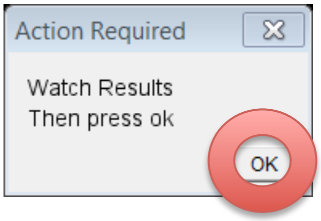

Figure 13 - Display after filtering.

3.12 After the calculation, few distorted frames can still be present depending on the chosen filtering method. To remove them, check the *Check to delete* box in the *Manual delete* window and press OK. If not, go to point **3.14**.

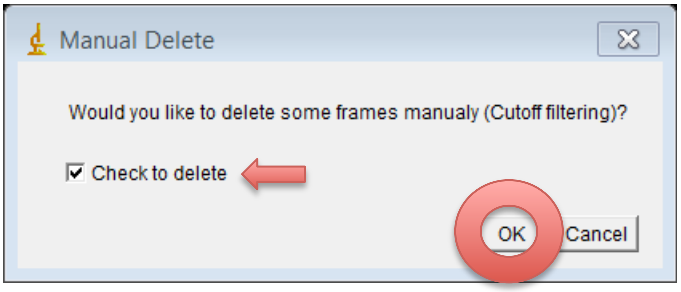

Figure 14 – Manual frame deletion (1).

3.13 In the new window, press the OK button to begin the manual deletion process. You will now be able to delete the remaining artifactual frames from the filtered sequence. Use the left and right arrows on the keyboard to slide between frames. Press the *Space Bar* to eliminate a frame (it will turn black). When you are done editing, press *Shift*.
Note: The deletion of a frame is irreversible, so proceed with caution.

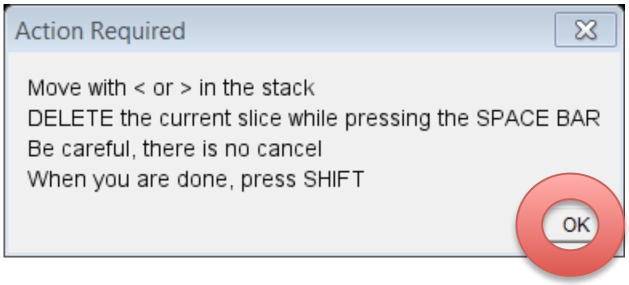

Figure 15 - Manual frame deletion (2).

3.14 Finally, you will be asked if you want to see all the results for each selected filtering method. When you are done, you can exit *ImageJ*. The results are available in *.avi* format encoded uncompressed at 7 frame per seconds and will be saved in the original image sequence folder, in the *Results* folder.

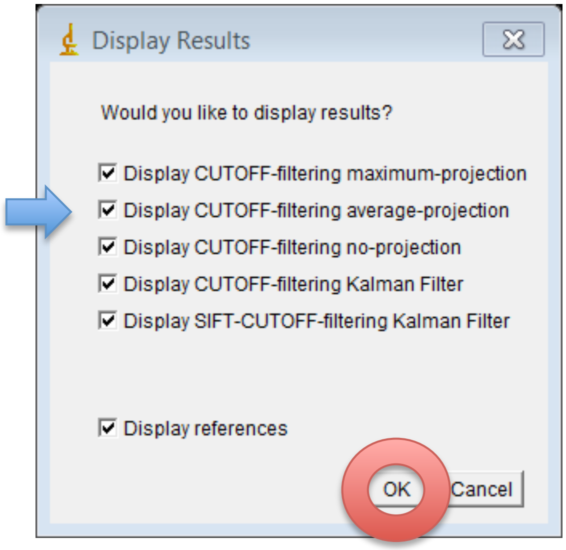

Figure 16 – Display final results

Table 1. Necessary plugins and their links.

Name	Link
StackReg and TurboReg	http://bigwww.epfl.ch/thevenaz/stackreg/
Kalman_Stack_Filter.class	http://rsbweb.nih.gov/ij/plugins/download/Kalman_Stack_Filter.class
Imagescience.jar	http://www.imagescience.org/meijering/software/transformj/
Jama-1.0.2.jar	http://math.nist.gov/javanumerics/jama/Jama-1.0.2.jar
Java_SIFT.jar	http://fly.mpi-cbg.de/~saalfeld/Projects/javasift.html
